# Genome-wide characterization of bZIP gene family identifies potential members involved in flavonoids biosynthesis in *Ginkgo biloba* L.

**DOI:** 10.1038/s41598-021-02839-2

**Published:** 2021-12-03

**Authors:** Huan Han, Feng Xu, Yuting Li, Li Yu, Mingyue Fu, Yongling Liao, Xiaoyan Yang, Weiwei Zhang, Jiabao Ye

**Affiliations:** 1 College of Horticulture and Gardening, Yangtze University, Jingzhou, 434025 Hubei China; 2 Hubei Key Laboratory of Economic Forest Germplasm Improvement and Resources Comprehensive Utilization, Hubei Collaborative Innovation Center for the Characteristic Resources Exploitation of Dabie Mountains, Huanggang Normal University, Huanggang, 438000 Hubei China

**Keywords:** Evolution, Molecular biology

## Abstract

*Ginkgo biloba* L. is an ancient relict plant with rich pharmacological activity and nutritional value, and its main physiologically active components are flavonoids and terpene lactones. The bZIP gene family is one of the largest gene families in plants and regulates many processes including pathogen defense, secondary metabolism, stress response, seed maturation, and flower development. In this study, genome-wide distribution of the bZIP transcription factors was screened from *G. biloba* database in silico analysis. A total of 40 bZIP genes were identified in *G. biloba* and were divided into 10 subclasses. *GbbZIP* members in the same group share a similar gene structure, number of introns and exons, and motif distribution. Analysis of tissue expression pattern based on transcriptome indicated that *GbbZIP08* and *GbbZIP15* were most highly expressed in mature leaf. And the expression level of *GbbZIP13* was high in all eight tissues. Correlation analysis and phylogenetic tree analysis suggested that *GbbZIP08* and *GbbZIP15* might be involved in the flavonoid biosynthesis. The transcriptional levels of 20 GbbZIP genes after SA, MeJA, and low temperature treatment were analyzed by qRT-PCR. The expression level of *GbbZIP08* was significantly upregulated under 4°C. Protein–protein interaction network analysis indicated that *GbbZIP09* might participate in seed germination by interacting with *GbbZIP32.* Based on transcriptome and degradome data, we found that 32 out of 117 miRNAs were annotated to 17 miRNA families. The results of this study may provide a theoretical foundation for the functional validation of GbbZIP genes in the future.

## Introduction

*Ginkgo biloba*, known as a “living fossil,” began to appear 280 million years ago and is widely distributed throughout the world^1^. *G. biloba* extract (EGb 761) is the initial drug of choice for Alzheimer’s treatment^2^. EGb is widely used in the treatment of hypertension, cardiovascular and neurological diseases^3–5^. The main physiologically active components of *G. biloba* extract are flavonoids and terpene lactones^6,7^. Flavonoids have free radical scavenging activity^8^. There are not less than 6% terpene trilactones and 24% flavonoids in EGb^9^. The demand for EGb in the market is increasing due to its important medicinal value. However, the content of flavonoid in *G. biloba* is extremely low which is a serious obstacle in the application of EGb. It has been reported that genetic engineering techniques is an effective way to enhance the content of secondary metabolites such as flavonoids^10^.

When plants are subjected to low temperature, drought, salt stress, or exogenous hormones, transcription factors are induced to bind to the corresponding cis-acting elements, activating the expression of downstream genes to improve plant tolerance^11^. bZIP, one of the largest families in transcription factor, plays an important role in physiological processes such as biotic and abiotic stress responses and development in plants^12^. All bZIP genes share a conserved bZIP domain, which contains two structural features (a DNA-binding basic region and a dimerizing leucine zipper region). The N-terminus contains a basic region of about 16 amino acid residues, including a nuclear localization signal, immediately followed by the N-X_7_-R/K conserved base sequence. The C terminus consists of a heptad repeat of leucine or other bulky hydrophobic amino acids^13,14^. To bind DNA, two subunits adhere via interaction between the hydrophobic sides of their helices, which creates a superimposing coiled-coil structure^15^. ACGT is the core sequence recognized by bZIP TF, and the corresponding cis-acting elements are A-box, G-box, and C-box^14,16^.

Flavonoid biosynthesis involved in multiple enzymes in plants, and its accumulation is not determined by a single key enzyme*.* Besides, the process is also regulated by many transcription factors. A bZIP transcription factor was found to be involved in the metabolism of flavonoids. *HY5* belongs to the H subclass of the bZIP gene family, which was proved to be involved in plant photomorphogenesis^17,18^. Subsequently, it was shown that *HY5* was also involved in the anthocyanin metabolism process, which belongs to flavonoids^19^. Qiu et al. obtained *SlHY5* frameshift mutated and found that the anthocyanin content in the mutant was lower than that in wild-type tomato^20^. *HY5* can improve plant cold tolerance through direct regulation of *CBF* or indirect regulation of *MYB15*^21^.Anthocyanins accumulation could be inhibited by Gibberellins at low temperature in plants, while the expression level of the gibberellin signaling pathway gene *GA2ox1* showed *HY5*-dependent^22^. In strawberry, *HY5*-*bHLH9* heterodimer formed to regulate light-dependent coloration and anthocyanin biosynthesis, suggesting that *HY5* can be involved in plant anthocyanin metabolism by acting together with other genes^23^. Nguyen et al.^24^ found *HY5* binds directly to the *MYBD* promoter in this process to promote anthocyanin accumulation. Zhang et al.^25^ found that *HY5* or *HYH* TFs promote anthocyanin accumulation by upregulating DFR, a key gene downstream of the anthocyanin biosynthesis pathway in *A*. *thaliana.* The bZIP TF was shown to affect the biosynthesis of anthocyanins in red raspberries^26^. Furthermore, the bZIP TF *HY5* in regulates anthocyanin synthesis through the activation of *AtMYB75* transcription in *A. thaliana*^27^. Droge et al.^28^ isolated the *G/HBF-1* (bZIP) TF from soybean and found that *G/HBF-1* can bind to the H-box and G-box in the promoter region of the CHS gene to activate CHS gene expression and substantially increase phytoalexin level, thereby improving disease resistance in soybean. Akagi et al.^29^ found that *DkbZIP5* was involved in proanthocyanidin biosynthesis by binding to the ABRE element of the promoter region of *DkMYB4* in persimmon callus. Fan et al.^15^ identified a total of 135 RsbZIP genes from radish genome and found that *RsbZIP011* and *RsbZIP102* were potential participants in the radish anthocyanin synthesis pathway. Therefore, regulating the expression of TFs is another effective way to increase flavonoid content.

The whole genome sequence of *G. biloba* has been published in 2016. The bZIP gene family is important in plant development and stress response. However, the bZIP transcription factors have not been reported in *G. biloba* yet. In the present study, the whole genome sequence of *G. biloba* was used to screen for the GbbZIP gene family. We analyzed the physicochemical property, phylogenetic relationship, chromosome localization, gene structure, motifs, tissue expression pattern, protein-protein interaction network, miRNAs of GbbZIP gene family and their expressional patterns under SA, MeJA, and low temperature treatment. The result will provide a theoretical basis for subsequent studies on the mechanism of action and function of the bZIP gene family in *G. biloba*.

## Results

### Identification of bZIP genes in *G. biloba*

**Figure 1:**
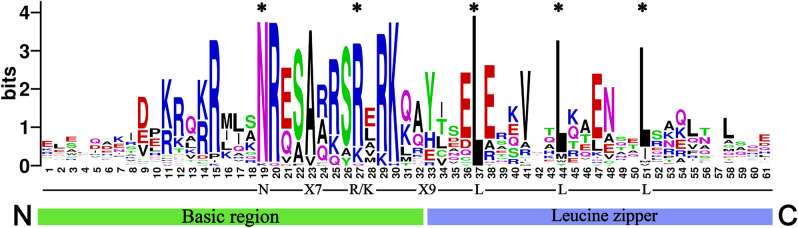
Visualization of GbbZIP family conserved domains in *G. biloba*. Alignment of GbbZIP conserved domains created using WebLogo program (http://weblogo.threeplusone.com/create.cgi). The total height of the letter piles at each position indicates the conservation of the sequence at that position (measured in bits). The height of a single letter in the letter piles represents the relative frequency of the corresponding amino acid at that position.

A total of 65 protein sequences were identified from the *G. biloba* genome using HMMER 3.0 software. 15 bZIP proteins were found to contain no bZIP domains or incomplete bZIP domains by further CDD and Pfam analysis, so they were subsequently removed. The remained 40 proteins containing bZIP domains were named *GbbZIP01*–*GbbZIP40*. The proteins encoded by the 40 GbbZIP genes ranged from 66 (*GbbZIP34*) to 681 (*GbbZIP22*) amino acids and from 7.90 kDa to 74.44 kDa in relative molecular weight. The isoelectric point was predicted to range from 5.16 to 9.75. As can be seen in Table 1, the GRAVY scores of 40 GbbZIP proteins were negative, indicating they are hydrophilic. 38 *GbbZIPs* are expressed in cell nucleus. *GbbZIP12* and *GbbZIP27* are located in the chloroplast and plastid, respectively. Multiple sequence alignment of the conserved domain sequences of 40 GbbZIP members showed that the basic region is composed of N-(X)7-R/K, and the zipper domain is composed of the heptapeptide repeat of leucine (L) or related hydrophobic amino acids (Fig. 1).

**Table 1:** The detailed information of GbbZIP family members.

Name	Gene ID	Chr	Protein size	pI	Mw	GRAVY	Subcellular location
*GbbZIP01*	Gb_07087	1	457	6.14	50.57	− 0.867	nucl
*GbbZIP02*	Gb_00122	1	303	5.16	34.92	− 0.836	nucl
*GbbZIP03*	Gb_07823	1	496	6.68	53.74	− 0.726	nucl
*GbbZIP04*	Gb_32028	1	455	6.69	48.04	− 0.788	nucl
*GbbZIP05*	Gb_29582	1	557	6.05	60.83	− 1.054	nucl
*GbbZIP06*	Gb_21161	2	199	7.72	21.87	− 0.78	nucl
*GbbZIP07*	Gb_06062	2	510	6.33	56.58	− 0.538	nucl
*GbbZIP08*	Gb_25822	3	154	8.67	47.58	− 1.274	nucl
*GbbZIP09*	Gb_29784	3	194	8.07	21.75	− 0.58	nucl
*GbbZIP10*	Gb_37770	4	369	8.96	40.37	− 0.822	nucl
*GbbZIP11*	Gb_28099	4	420	6.95	46.66	− 0.479	nucl
*GbbZIP12*	Gb_28107	4	190	9.01	22.06	− 0.472	chlo
*GbbZIP13*	Gb_16382	4	475	9.54	50.24	− 0.524	nucl
*GbbZIP14*	Gb_35962	4	253	8.76	27.42	− 0.691	nucl
*GbbZIP15*	Gb_12012	5	278	7.66	31.20	− 0.864	nucl
*GbbZIP16*	Gb_12023	5	269	7.22	29.48	− 0.683	nucl
*GbbZIP17*	Gb_34968	6	448	8.27	49.81	− 0.705	nucl
*GbbZIP18*	Gb_38174	6	437	9.24	48.58	− 0.838	nucl
*GbbZIP19*	Gb_16507	6	648	6.21	71.22	− 0.893	nucl
*GbbZIP20*	Gb_32471	7	275	5.62	30.63	− 0.738	nucl
*GbbZIP21*	Gb_24798	7	389	6.38	43.57	− 0.817	nucl
*GbbZIP22*	Gb_26079	7	681	6.16	74.44	− 0.878	nucl
*GbbZIP23*	Gb_00618	8	269	7.97	30.40	− 0.595	nucl
*GbbZIP24*	Gb_05158	8	326	9.27	36.45	− 0.674	nucl
*GbbZIP25*	Gb_11735	8	306	9.15	34.05	− 0.68	nucl
*GbbZIP26*	Gb_20332	8	419	9.6	46.43	− 0.536	nucl
*GbbZIP27*	Gb_05253	8	645	6.8	72.92	− 0.504	plas
*GbbZIP28*	Gb_30625	8	285	6.6	30.88	− 0.681	nucl
*GbbZIP29*	Gb_16168	9	466	9.56	50.02	− 0.634	nucl
*GbbZIP30*	Gb_03180	9	474	8.6	52.04	− 0.559	nucl
*GbbZIP31*	Gb_41342	9	317	5.95	35.56	− 0.544	nucl
*GbbZIP32*	Gb_20759	9	219	8.74	25.31	− 0.741	nucl
*GbbZIP33*	Gb_40580	9	468	7.76	49.76	− 0.765	nucl
*GbbZIP34*	Gb_21753	10	66	9.75	7.90	− 1.639	nucl
*GbbZIP35*	Gb_15846	10	435	8.16	48.64	− 0.664	nucl
*GbbZIP36*	Gb_10432	10	228	9.17	61.90	− 0.691	nucl
*GbbZIP37*	Gb_13495	11	464	5.81	48.70	− 0.725	nucl
*GbbZIP38*	Gb_33985	12	206	8.53	23.78	− 0.824	nucl
*GbbZIP39*	Gb_20987	12	365	6.49	40.59	− 0.885	nucl
*GbbZIP40*	Gb_21347	12	503	9.21	53.29	− 0.492	nucl

### Chromosomal localization and phylogenetic analysis

**Figure 2:**
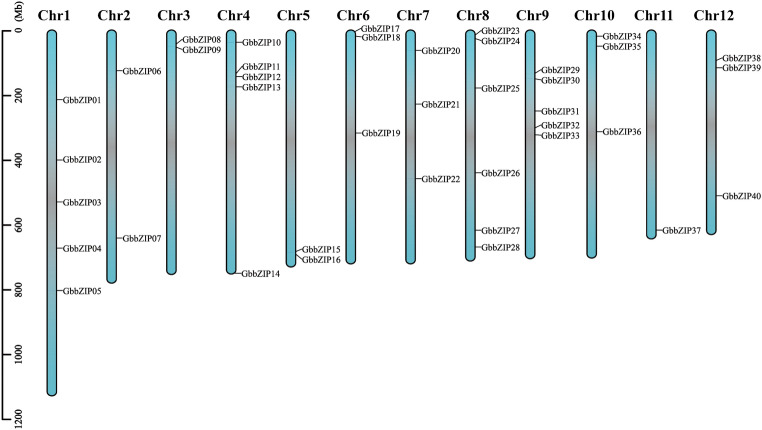
Chromosome localization and distribution of *G. biloba* bZIP genes. The scale on the left is in megabases (Mb).

Based on genome annotation data, the chromosomal distribution of GbbZIP genes was visualized using TBtools v1.09854 in *G. biloba*. The bZIP genes are distributed unevenly on 12 chromosomes of *G. biloba*. A total of 5, 2, 2, 5, 2, 3, 3, 6, 5, 3, 1, and 3 GbbZIPs were distributed on Chr1–Chr12, respectively. Fig. 2 shows that Chr11 contains the fewest GbbZIP genes with only 1, while Chr8 contains the most genes with 6.

**Figure 3:**
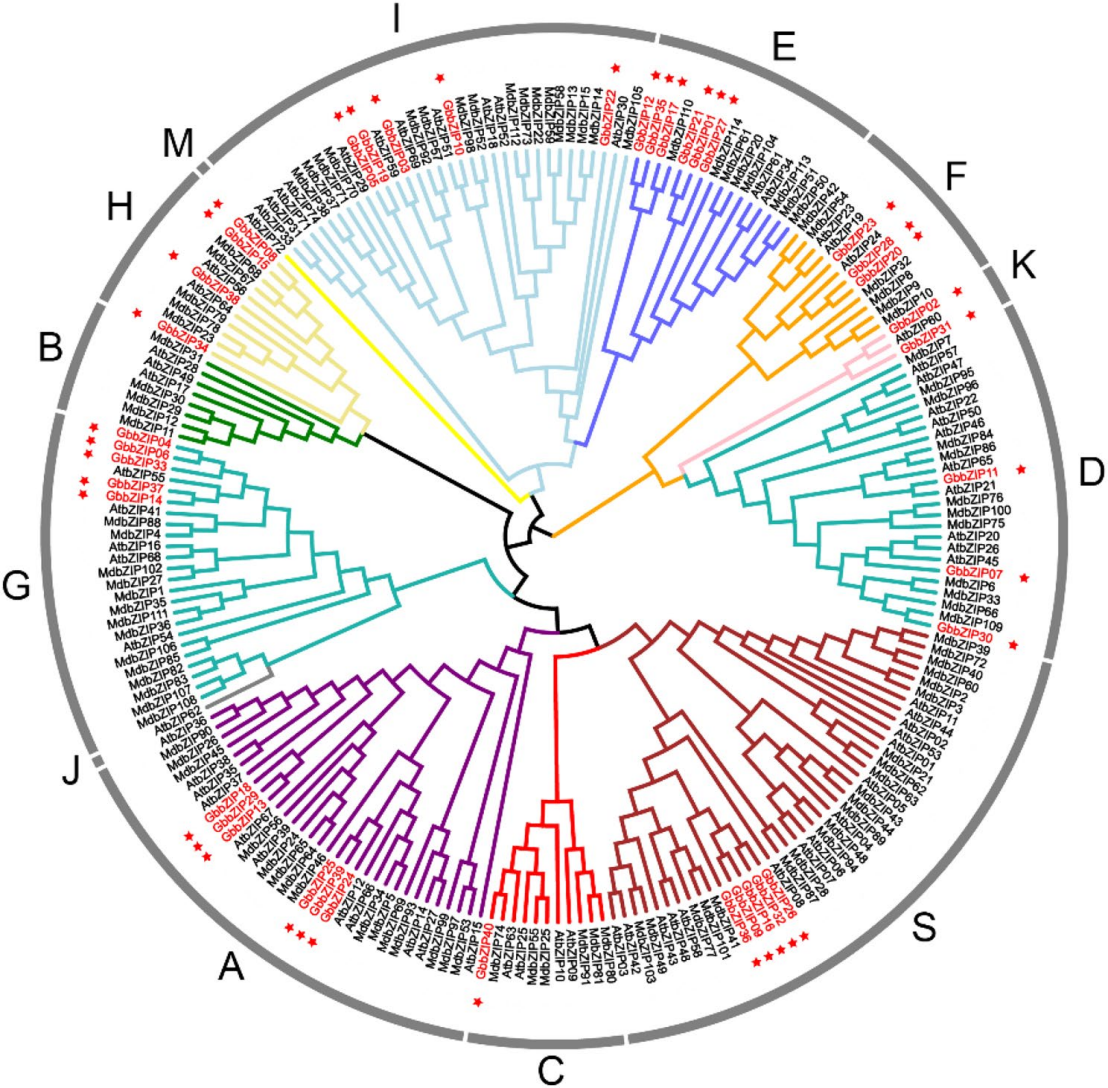
Phylogenetic tree based on *G. biloba*, *A. thaliana* and *M. domestica* bZIP proteins. MUSCLE in MEGA 6.0 was used for multiple sequence alignments of bZIP conserved domain sequences^31,32^. The neighbor-joining phylogenetic tree was constructed using MEGA 6.0 with the following parameters: p-distance, pairwise deletion and bootstrap (1000 replicates). The red stars represent GbbZIP members in *G. biloba.*

To investigate the phylogenetic relationship among the bZIP family members, a phylogenetic tree was generated including 69 AtbZIPs in *A*. *thaliana* and 109 MdbZIPs in *Malus domestica*. Based on the previous classification of 69 AtbZIPs and 109 MdbZIPs members^30^, 40 GbbZIPs were divided into 10 subfamilies (Fig. 3): A (6), B (0), C (1), D (3), E (6), F (3), G (5), H (4), I (5), S (5), J (0), K (2), and M (0). In *G. biloba*, group B, J, and M don't contain GbbZIP members. Group A and E are the largest subfamilies while group S is the largest in *A*. *thaliana* and *M. domestica*.

### Gene structure and motif analysis

**Figure 4:**
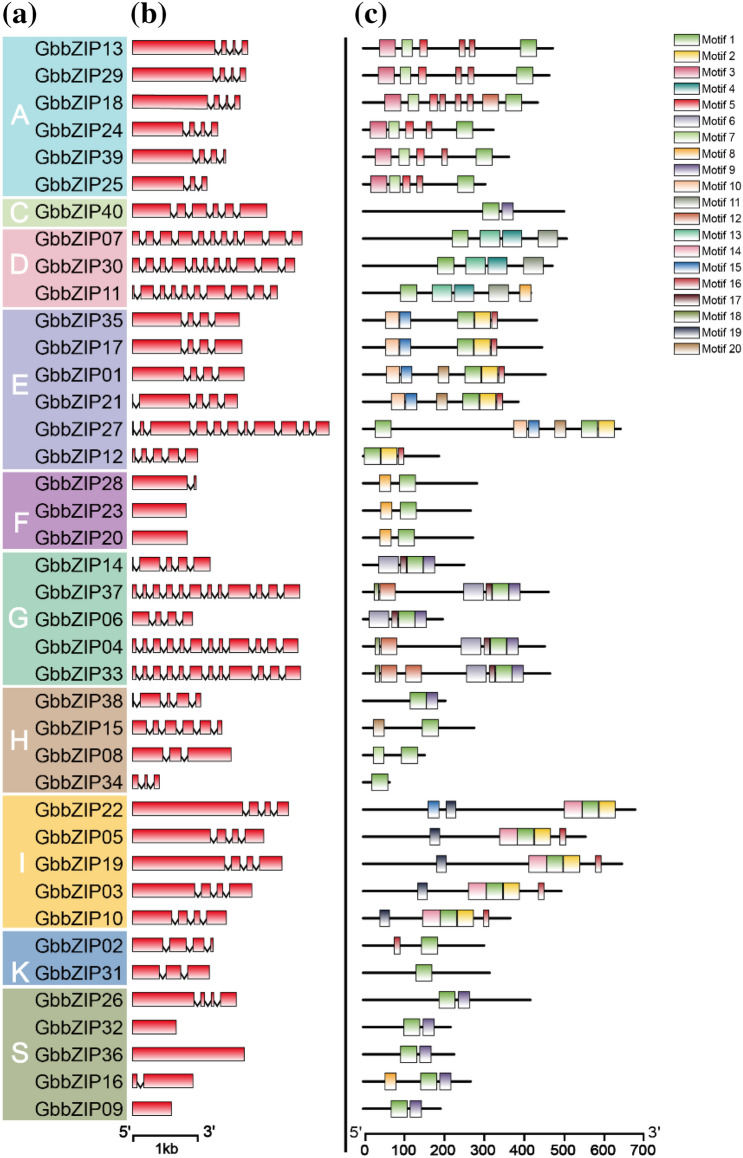
Characterization of GbbZIP genes in *G. biloba*. (**a**) The clustering of GbbZIP proteins based on Neighbor-Joining phylogenetic tree. (**b**) Exon–intron structures of GbbZIP genes were visualized with GSDS website (http://gsds.gao-lab.org/). The red block represents the coding sequence (CDS), the broken line represents intron. (**c**) Distribution of conserved motifs for GbbZIP proteins was visualized with TBtools v1.09854.

To gain insights into the structures of GbbZIP genes, their exon/intron organization was investigated. As shown in Fig. 4b, the number of exons in GbbZIPs ranged from 1 to 12. The gene structure of the same subfamily was relatively conserved. For example, four members in group E contained the same number and length of exons. Out of the six members in group A, five are composed of a long exon, and three short exons. A higher number of exons were found in D and G subclasses, while fewer introns were found in F and S subclasses. The gene structure of subclass A was highly uniform, but the number of introns in subclass H varied considerably.

The motifs of GbbZIPs were analyzed using the MEME website and the results were shown in Fig. 4c. A total of 20 motifs were found in GbbZIPs. Using the PFAM database, motif 1 and motif 9 (Table S2) were identified as the core conserved domains of GbbZIP proteins. Motif 13 and 4 were identified as DOG1 (Delay of Germination 1), which appears to be a highly specific controller seed dormancy^33^. Motif 12 and 18 were identified as MFMR (Multifunctional mosaic region), and other motifs contained no specific annotation information^34^. In addition to the core conserved domains, different conserved motifs were also present in different subfamilies. For example, Motif 3, 5, and 7 are only present in subfamily A; motif 4, 11, and 13 are distributed in subfamily D; motif 6, 12, 15, 17, and 18 exist in subfamily G; motifs 8 and motif 10 occur in subfamilies F and E, respectively. Motif 9 was detected only in subclass G and S, suggesting similar functions between the two subclasses that were in the same branch of the evolutionary tree.

### Promoter analysis

**Figure 5:**
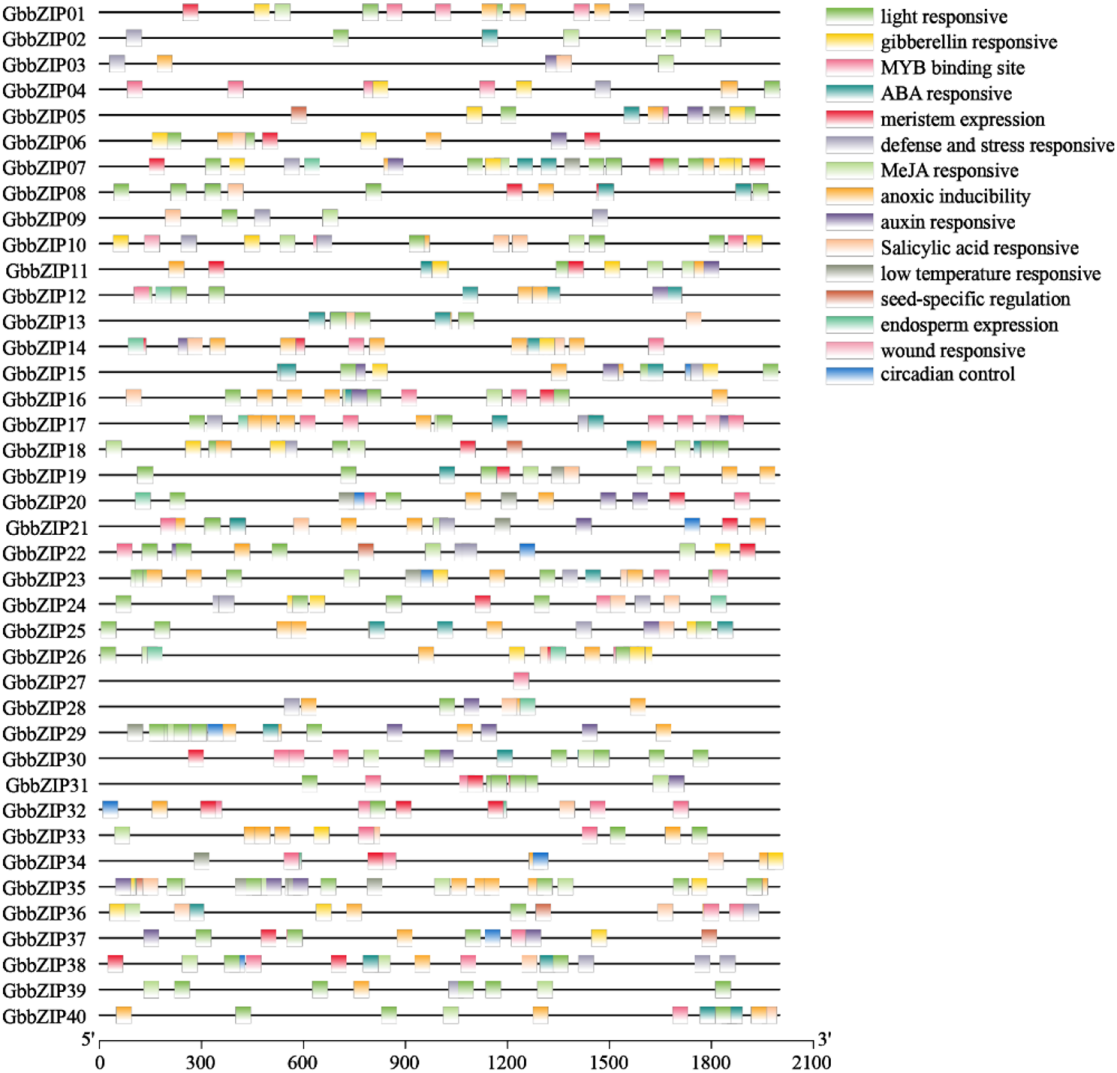
Cis-acting elements on the promoters of *G. biloba* bZIP genes. The 2000 bp upstream sequences of the transcription start site in GbbZIPs were extracted from the *G. biloba* genome database, and their promoter sequences were submitted to the PlantCARE website (http://bioinformatics.psb.ugent.be/webtools/plantcare/html/).

The sequence 2000 bp upstream of each GbbZIP gene was analysed in PlantCARE website, and the results were visualized with TBtools v1.09854. The detailed cis-acting element information can be found in Table S3. As shown in Fig. 5, the most frequently occurring cis-acting elements contain light-, hormone-, abiotic stress-, and growth- and development- responsive element. G-box, ACE, and GT1 motifs are light-responsive elements, while ABRE, TGA, CGTCA-motif, and TCA-element are ABA-, auxin-, MeJA-, and SA-responsive elements, respectively. Gibberellin responsive element contains GARE-motif and P-box. Among them, the light-responsive element appeared most frequently (158 times) and was the most widely distributed in GbbZIP gene promoters. Nine GbbZIPs contained low-temperature-responsive elements. In addition, other elements include anoxic inducibility (ARE), circadian control, defense and stress (TC-rich), endosperm expression (GCN4-motif), and meristem expression (CAT-box) elements.

### Analysis of the expression pattern and correlation

**Figure 6:**
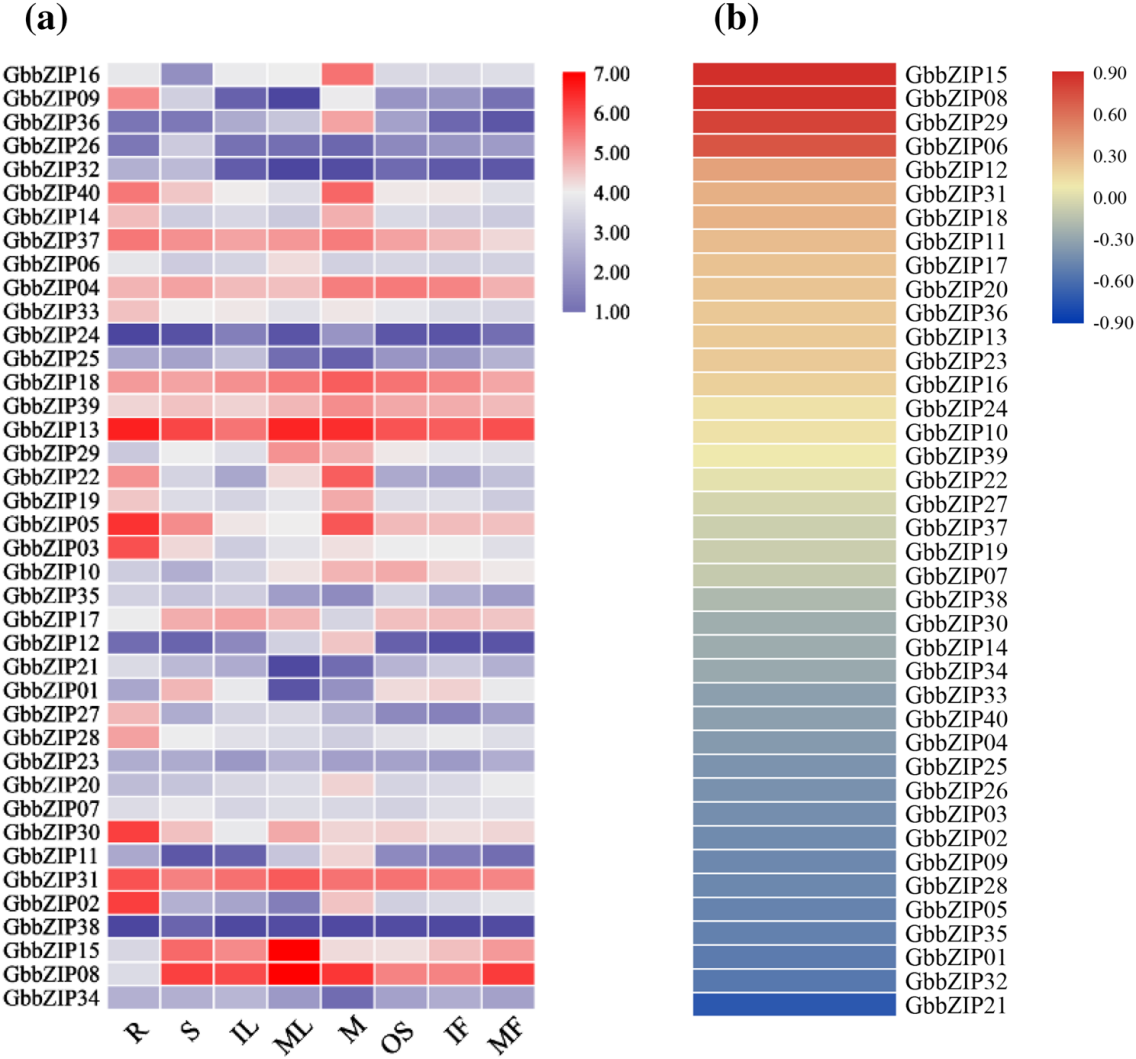
(**a**) Expression pattern of GbbZIPs in the 8 tissues base on the FPKM in the transcriptome data. (*R* root, *S* stem, *IL* immature leaf, *ML* mature leaf, *M* microstrobilus, *OS* ovulate strobilus, *IF* immature fruit, *MF* mature fruit). (**b**) Correlation analysis (p < 0.05) of the content of flavonoid and the FPKM of GbbZIPs in 8 tissues base on the FPKM in the transcriptome data.

The expression pattern of the GbbZIP gene family was analyzed based on FPKM from *G. biloba* transcription database. As shown in Fig. 6a, 36 GbbZIPs are expressed in eight tissues, except for four genes (*GbbZIP09*, *GbbZIP24*, *GbbZIP32,* and *GbbZIP38*) that are not expressed in mature leaf and root. *GbbZIP13*, *GbbZIP18*, and *GbbZIP31* showed high expression characteristics in all eight tissues. *GbbZIP38*, *GbbZIP24*, *GbbZIP26*, and *GbbZIP32* showed very low expression levels in all tissues. The expression profiles of *GbbZIP11*, *GbbZIP12*, *GbbZIP16*, and *GbbZIP36* were significantly higher in microstrobilus than in other tissues. The expression levels of *GbbZIP01*, *GbbZIP02*, *GbbZIP21*, and *GbbZIP25* were lower in mature leaf than in other tissues.

Based on correlation analysis between on the FPKM of GbbZIPs and the flavonoid content in the eight tissues of *G. biloba*, we obtained two GbbZIPs (*GbbZIP08* and *GbbZIP15*) with correlation coefficients greater than 0.8 (Fig. 6b, Table S4, Table S6, Table S7). These two genes were assigned to subclass H in the evolutionary tree analysis.

### GbbZIP protein–protein interaction network prediction

**Figure 7:**
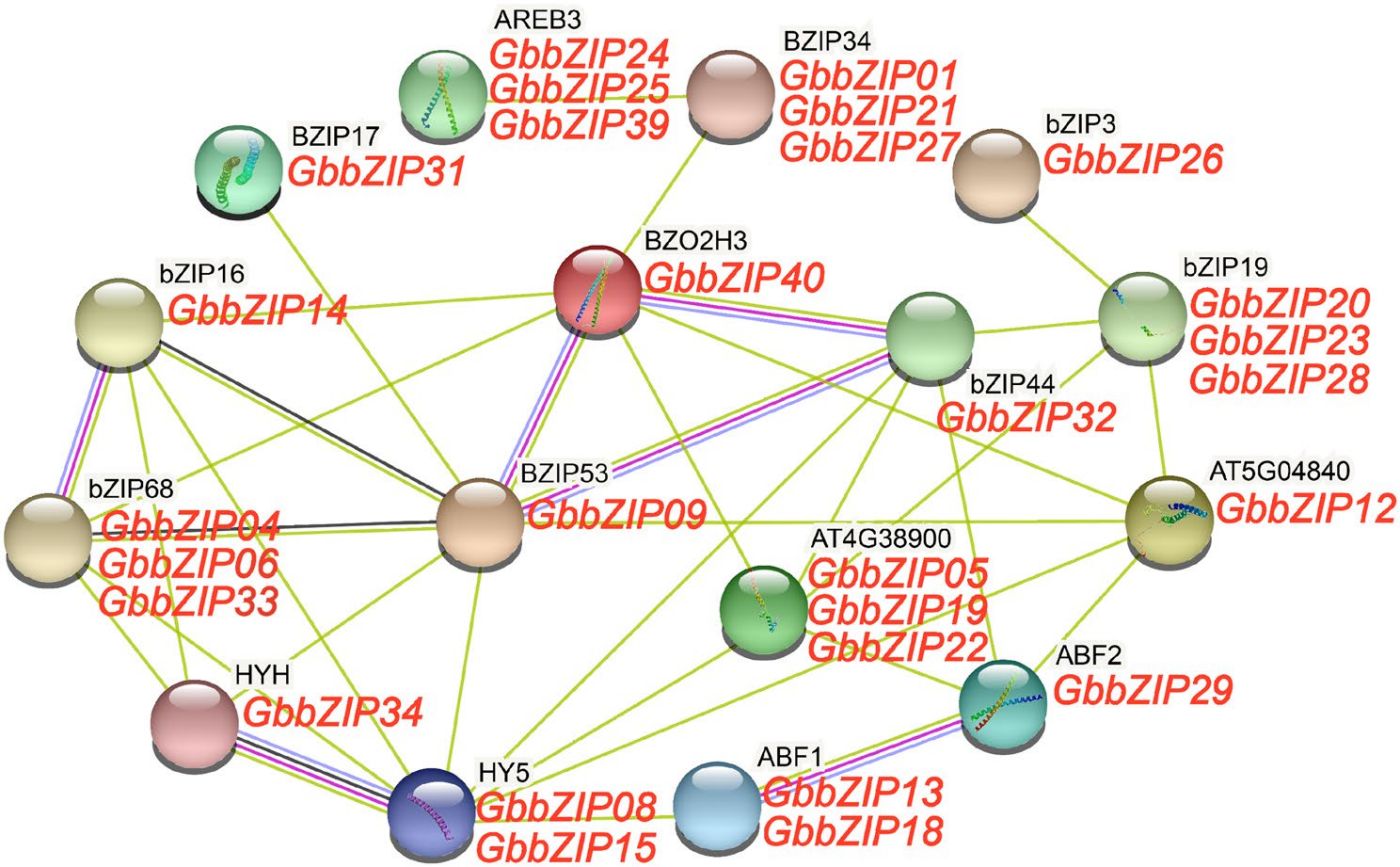
Protein–protein interaction network for GbbZIPs was analyzed by the STRING website (http://string-db.org/) using the full-length protein sequence^16,36^. Empty nodes indicate proteins of unknown 3D structure. Filled nodes indicate that some 3D structure is known or predicted.

The interaction between GbbZIP proteins was predicted in the website STRING. 33 out of 40 GbbZIP proteins were on the protein-protein interaction network. As shown in Fig. 7, *AREB3* (AT3G56850) binds to the embryo specification element and the ABA-responsive element (ABRE) and participates in abscisic acid-regulated gene expression during seed development. *ABF2* (AT1G45249) binds to the ABRE motif and involves in drought tolerance. *HY5* (AT5G11260) and *HYH* (AT3G17609) are transcription factors that promote photomorphogenesis in light. Plants inhibit anthocyanin biosynthesis by degrading *HY5* at high temperature^35^.

### Prediction and analysis of miRNA regulating GbbZIPs

**Figure 8:**
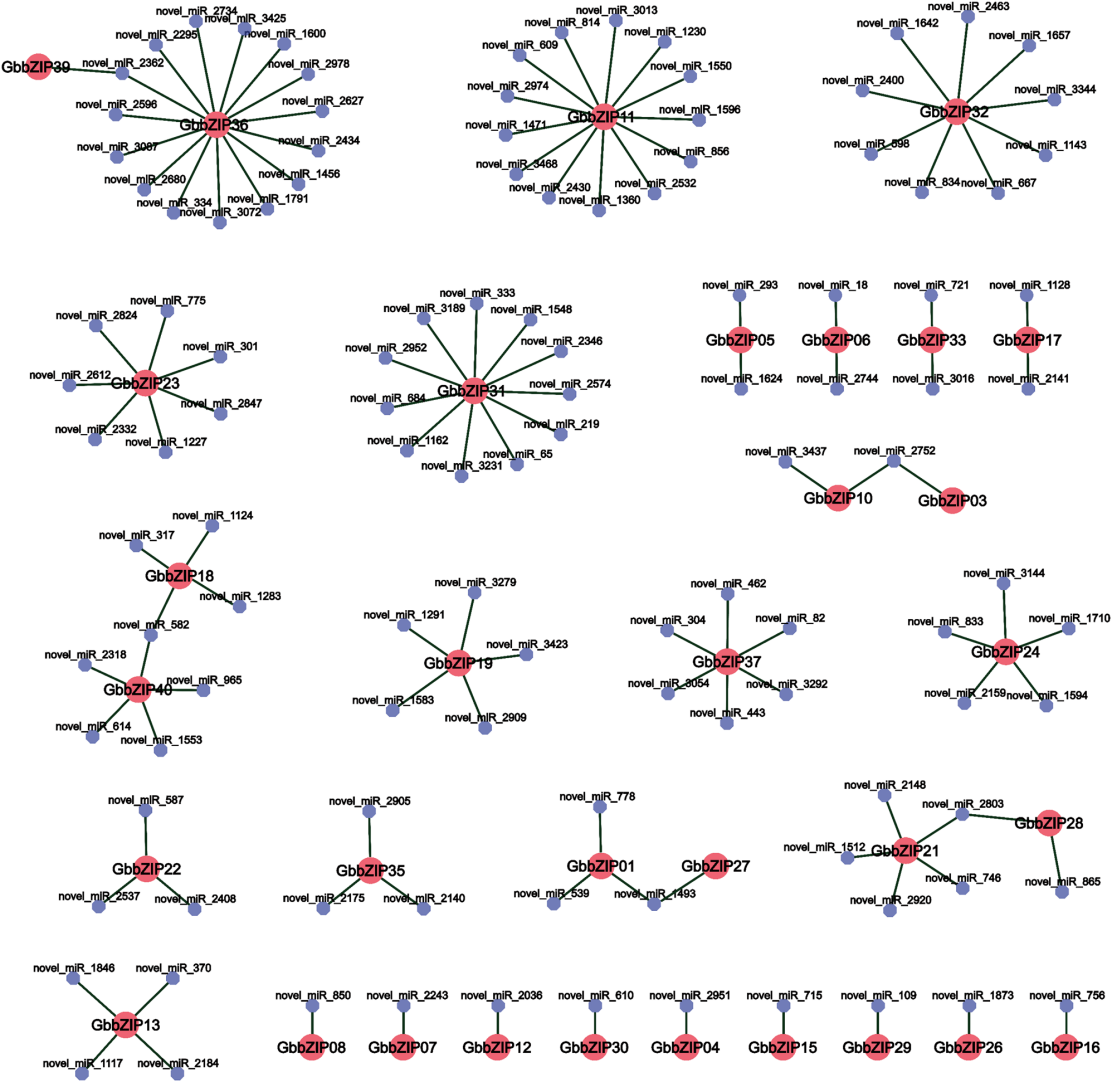
Network diagram of miRNAs targeting GbbZIPs in *G. biloba* was created using the OmicShare tools (http://www.omicshare.com/tools)*.* Red circle represents GbbZIP members. lilac circle represents miRNAs. The data for the network diagram were obtained from transcriptome and degradome sequencing data.

In order to further understand the GbbZIP genes in *G. biloba*, we combined transcriptome and degradome sequencing results to screen for 117 miRNA members regulating 33 GbbZIPs. Based on the transcriptome annotation results, a total of 32 miRNAs were matched to 17 miRNA families (Table S5). Fig. 8 showed that 9 out of 40 GbbZIPs were each targeted by only one corresponding miRNA. *GbbZIP36*, *GbbZIP31*, *GbbZIP11*, and *GbbZIP32* were targeted by 15, 11, 13, and 9 miRNAs, respectively. Interestingly, some miRNAs targeted more than one GbbZIP gene, such as novel_miR_582 (*GbbZIP18* and *GbbZIP40*), novel_miR_2803 (*GbbZIP21* and *GbbZIP28*), novel_miR_2362 (*GbbZIP36* and *GbbZIP39*), novel_miR_1493 (*GbbZIP01* and *GbbZIP27*), and novel_miR_2752 (*GbbZIP03* and *GbbZIP10*). *GbbZIP08* and *GbbZIP15* were targeted by novel_miR_850 and novel_miR_715, respectively.

### Differential expression levels of GbbZIPs with hormone treatment

**Figure 9:**
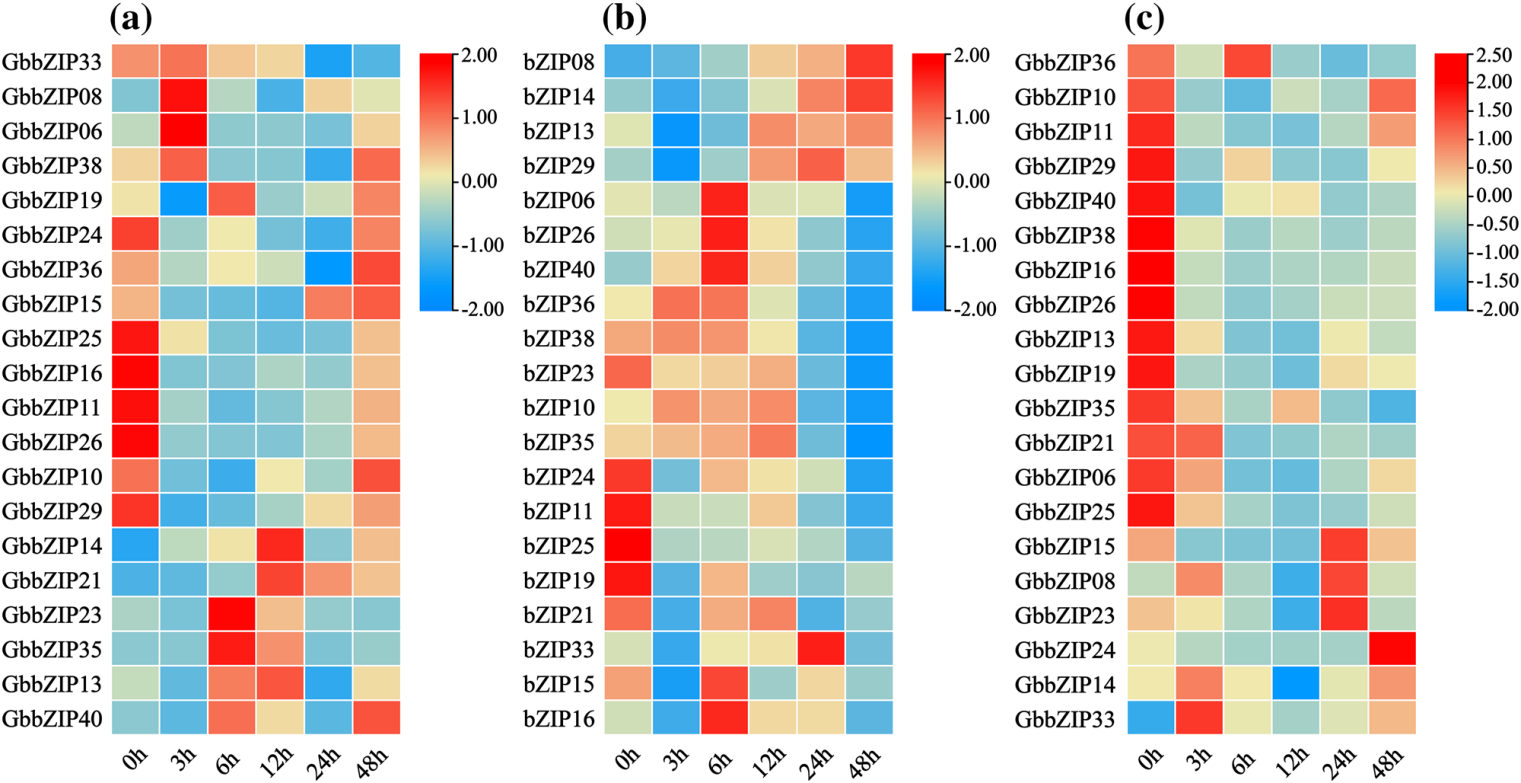
Heat maps of the relative expression of GbbZIPs under abiotic stress. (**a**) The relative expression of GbbZIPs under MeJA. (**b**) The relative expression of GbbZIPs under cold stress. (**c**) The relative expression of GbbZIPs under SA. Expression values are mapped to a color gradient from low (blue) to high (red) expression.

Based on the results of cis-acting element analysis, genes containing a large number of cis-acting elements which are related to SA-, MeJA-, and low temperature-responses were selected for qRT-PCR analysis, and the results were visualized in the form of a heatmap. As shown in the Fig. 9c, most GbbZIPs responded negatively to SA, only the expression levels of *GbbZIP08* and *GbbZIP33* first increased and then decreased. *GbbZIP25*, *GbbZIP16*, *GbbZIP11*, *GbbZIP11*, *GbbZIP26*, *GbbZIP10*, and *GbbZIP29* were downregulated within 24 h after MeJA treatment then increased at 48 h (Fig. 9a). The expression levels of *GbbZIP23* and *GbbZIP35* peaked at 6 h after MeJA treatment and then started to decrease (Fig. 9a). With low-temperature treatment, the transcription levels of *GbbZIP08* and *GbbZIP14* were upregulated, while *GbbZIP26* increased and then decreased, and reached a peak at 6 h (Fig. 9b).

## Discussion

*G. biloba* is an ancient economic tree species that originated in China and it has a very strong ability to adapt to harsh environments. Therefore, the genetic information of *G. biloba* is of great research value in plant growth and development, pest and disease defense, abiotic stress, and tree evolution. To date, the bZIP gene family has been identified in *A. thaliana* (78 bZIPs)^30^, *Populus* (86 bZIPs)^37^, *Raphanus sativus* (135 bZIPs)^15^, *Solanum tuberosum* (80 bZIPs)^38^, *Brassica napus* (247 bZIPs)^38^, and *Olea europaea* (103 bZIPs)^39^. However, the bZIP transcription factors in *G. biloba* have not been reported yet. In this study, a total of 40 GbbZIP members were identified based on the genomic data of *G. biloba*. Compared to the species above, *G. biloba* contains the fewest bZIP members with 40 while *B. napus* contains the most with 247.

We used phylogenetic analysis to classify bZIP genes into 10 groups—40 GbbZIP genes, along with 69 bZIP genes from *A. thaliana*, and 109 from *M. domestica* (Fig. 3)*.* The GbbZIP members were not detected in subgroup J, B, and M, which implying that these members were derived gradually during the evolutionary process. In *G. biloba*, the largest number of GbbZIPs were found in subclasses S, A, and I subgroups. It is similar to *A. thaliana*^30^, *Populus*^37^, *R. sativu*^15^, *S. tuberosum*^40^, *B. napus*^38^, and *O. europaea*^39^ which contains the most bZIP members in S, A, I, and D subclasses. In the Fig. 3, *GbbZIP03* was clustered closely with *AtbZIP51* (VIP1), which is a putative host factor in Agrobacterium-mediated T-DNA transfer^41^. Furthermore, GbbZIPs in the same group share a similar gene structure, number of introns and exons, and motif distribution.

HY5 (Elongated Hypocotyl 5) is a major regulator of photomorphogenesis. Studies in apple indicated that *MdHY5* promoted anthocyanin accumulation by regulating the expression of *MdMYB10* and downstream anthocyanin biosynthesis genes^42^. *SlHY5* in *Solanum lycopersicum* could directly recognize and bind to the G‐box and ACGT‐containing element in the promoters of anthocyanin biosynthesis genes, such as *CHS1*, *CHS2*, and *HDR*^43^. It is apparent in the Fig. 6b, the positive correlation between the expression of *GbbZIP15* and *GbbZIP08* and the content of flavonoids was the highest. In addition, *GbbZIP15* and *GbbZIP08* was close to *AtbZIP56* and *MdbZIP67* in the phylogenetic analysis. Blast 2.7.1, a local BLAST program, was used to search for protein sequences similar to *GbbZIP08* and *GbbZIP15* in the *A. thaliana* and *M. domestica* bZIP protein database. *AtbZIP56* and *MdbZIP67* proteins were found to be the most similar to *GbbZIP08* and *GbbZIP15* proteins (Table S8, Table S9). *AtbZIP56* (*HY5*) in subclass H has been reported to play an important role in anthocyanin biosynthesis^24^. *MdWRKY11* transcription factor was reported to bind to the W-box in the *MdbZIP67* promoter and thus participated in the anthocyanin biosynthesis in *M. domestica*^44^. The result implied that *GbbZIP15* and *GbbZIP08* might be involved in anthocyanin biosynthesis of *G. biloba*.

SA is a crucial internal signaling molecule needed for the induction of plant defense responses upon attack of a variety of pathogens^45^. The expression of *GbbZIP33* was increased by 20-fold 3 h after SA treatment. However, no significant changes were observed in the expression of *GbbZIP33* after MeJA and low-temperature treatment. In Cassava, *MebZIP3* was largely increased while *MebZIP5* expression was decreased after SA treatment^46^. The trend of *GbbZIP08* was relatively consistent in MeJA and SA treatment. Its expression level first increased, then decreased, and peaked 3 h after treatment. It showed a similar trend with *CsBZIP40* in sweet orange. The expression level of *CsBZIP40* increased significantly from 12 h and then decreased after 36 h After MeJA treatment^47^. With low-temperature treatment, *GbbZIP08* showed an increasing trend from 0 h to 8 h, and the expression was significantly increased. Similarly, the result was also found in apple. *MdbZIP67 (MdHY5)* overexpression improved the cold resistance in apple and transgenic *A. thaliana*^48^. It indicated that *GbbZIP08* might participate in the cold stress in *G. biloba*. The expression level of *GbbZIP29* was upregulated under low-temperature stress while the low-temperature-responsive element (CCGAAA) was found in *GbbZIP29* In the promoter analysis.

In the protein-protein interaction analysis, it revealed that 33 out 40 GbbZIP members were on the protein-protein interaction network (Fig. 7). *BZIP53* (AT3G62420) and *bZIP44* (AT1G75390) both involved in the positive regulation of seed germination through *MAN7* gene activation^49^. Therefore, the interaction between *BZIP53* (homology of *GbbZIP09*) and *bZIP44* (homology of *GbbZIP32*) might participate in seed germination. *GbbZIP08* and *GbbZIP15* were speculated as homologous genes of *HY5* (*AtbZIP56*). It has been reported that *HY5* was involved in modulating seed germination and seedling development in response to abscisic acid and salinity^50^. *HYH* (*HY5* homology) has a similar functions as *HY5* in plant photomorphogenesis and anthocyanin accumulation^22,51^. It was speculated that *GbbZIP34* (homology of *HYH*) was involved in flavonoid biosynthesis in *G. biloba* by interacting with *GbbZIP08* and *GbbZIP15* (homology of *HY5*).

The reported miRNAs associated with anthocyanin biosynthesis include miR858a, miR828, and miR156. In tomato, miR828 repressed anthocyanin expression by cleaving the structural gene *SlMYB7-like*^52^. 32 out of 117 miRNAs were annotated to 17 families (Table S5). In this study, *GbbZIP08* and *GbbZIP15* were targeted by novel_miR_850 and novel_miR_715, respectively. The miRNAs (novel_miR_1493, novel_miR_30, novel_miR_3054, novel_miR_3292, novel_miR_443, novel_miR_462, and novel_miR_82) were annotated as miR1314 family and they all targeted *GbbZIP37*. MIR1314 appears gymnosperm-specific and is likely to exist as a luster^53^. *GbbZIP06* was targeted by novel_miR_1493 and novel_miR_18 which were identified as miR397. Overexpression of native Musa-miR397 enhanced plant biomass without compromising abiotic stress tolerance in banana^54^. The miRNAs (novel_miR_2243, novel_miR_3072, novel_miR_2847, and novel_miR_775) were annotated as miR159 in this study. It was reported that miR159 inhibited the root growth by repressed the expression of *MYB33*, *MYB65* and *MYB101* in *A. thaliana*^55^.

## Conclusions

In the current study, the genome-wide distribution of the GbbZIP gene family was identified in *G. biloba*. We conducted a preliminary analysis of the physicochemical properties, chromosome localization, evolutionary relationship, tissue-specific expression pattern, protein–protein interaction, and miRNAs. We verified the response of 20 GbbZIPs to MeJA, SA, and low temperature through qRT-PCR. Based on the correlation analysis and the phylogenetic analysis, *GbbZIP08* and *GbbZIP15* were inferred to be *HY5* genes involved in anthocyanin biosynthesis in *G. biloba*. The results will be important for the further functional characterization of *GbbZIP08* and *GbbZIP15* in *G. biloba*.

## Methods

### Plant materials and treatments

Annual *G. biloba* seedlings were used in this study. The *G. biloba* seeds were derived from Ginkgo Resource Garden at the western campus of Yangtze University (E:112.15373°, N:30.357146°) and they were carried out in accordance with relevant institutional, national or international guidelines and regulation. Before sowing, *G. biloba* seeds were immersed in tap water for 3 days to absorb enough water. Then, they were taken out and covered with wet gauze to be promoted germination. When the radicle of the seeds grew to 1 cm, they were planted in pots (15 cm in height and 17 cm in diameter) and grown in a greenhouse (E:112.152283°, N:30.358268°). We watered the seedlings every 5 days. When the seedlings developed 5–6 leaves after 2 month, the leaves of seedlings were treated with 10 mM salicylic acid (SA), 1 mM methyl jasmonate (MeJA) and 4 °C, respectively^56,57^. Exogenous hormones were sprayed on the seedling leaves in this study. Leaves treated with water were used as the control. Three replicates were selected for each treatment, and each replicate contained 10 seedlings. After three different treatments, leaves of annual *G. biloba* seedlings in each treatment were collected at 0, 3, 6, 12, 24, and 48 h, separately^58^.

### Identification of GbbZIP genes

The genome data and annotated files of *G. biloba* were downloaded from the GIGA Database website (http://gigadb.org/dataset/100613)^59,60^. The hidden Markov model (PF00170, PF07716, and PF03131) of the bZIP gene was obtained from the Pfam database^61^. Protein sequences containing the bZIP domain were searched in the *G. biloba* protein database using HMMER 3.0 software. Subsequently, CDD (https://www.ncbi.nlm.nih.gov/Structure/cdd/wrpsb.cgi)^62^ and SMART (http://smart.embl-heidelberg.de/)^63^ searches were used to check the integrity of the bZIP domain. The conserved domain sequence of each GbbZIP member was extracted and used for multiple sequence alignment with MEGA 6.0 software. The alignment file was visualized in the website WebLogo (http://weblogo.threeplusone.com/create.cgi). The physicochemical properties of GbbZIP proteins were predicted using the online website ExPASy^64^. The website WoLF PSORT (https://wolfpsort.hgc.jp/) was used to predict subcellular localization of GbbZIPs.

### Phylogenetic analysis and chromosome localization

All bZIP protein sequences of *A. thaliana* and *M. domestica* were downloaded from the TAIR database (https://www.arabidopsis.org/index.jsp)^48^ and GDR database (https://www.rosaceae.org/)^65^, respectively. Multiple sequence alignment of bZIP proteins was performed using MUSCLE in MEGA 6.0^66^. The neighbor-joining phylogenetic tree was then constructed using MEGA 6.0 with the following parameters: p-distance, pairwise deletion, and bootstrap (1000 replicates)^67^. Combining the genome sequence files and annotation files, gene chromosome localization was visualized using the TBtools v1.09854^68^.

### Analysis of gene structure, conserved motif, and promoter

To further analyze the distribution of the GbbZIP genes in *G. biloba*, GbbZIP protein motifs were predicted on the website MEME (https://meme-suite.org/meme/)^4^. To acquire the gene structure, the nucleus sequences of GbbZIP genes were submitted to GSDS website (http://gsds.gao-lab.org/)^69^. The 2000 bp upstream sequences of the transcription start site in GbbZIPs were extracted from the *G. biloba* genome database, and their promoters were predicted on the PlantCARE website (http://bioinformatics.psb.ugent.be/webtools/plantcare/html/)^70^.

### Expression pattern analysis based on transcriptome and correlation analysis

To investigate the expression pattern of the GbbZIP gene family, we used FPKM of GbbZIP genes in eight tissues (root, stem, immature leaf, mature leaf, microstrobilus, ovulate strobilus, immature fruit, and mature fruit) based on the transcriptome data^71^. Subsequently, the correlation index between the FPKM of GbbZIP genes and the content of flavonoid in eight tissues was calculated (p < 0.05) by Pearson's correlation method using the OmicShare tools (http://www.omicshare.com/tools).

### Protein–protein interaction network

The protein sequences of 40 GbbZIP genes were extracted and sent to the STRING website (http://string-db.org/). The interaction network of GbbZIP proteins was predicted in the STRING website using a model plant *A. thaliana*^16,72^. The result was imported into Adobe Illustrator 2020 software for modification.

### miRNA target prediction of GbbZIP genes

Based on transcriptome and degradation data, miRNAs targeting the GbbZIP genes were screened from the annotation files^6,71^. The screening results were visualized using OmicShareTools (http://www.omicshare.com/tools), a free online platform.

### qRT-PCR analysis

Total RNA of *G. biloba* was extracted using the MiniBEST Plant RNA Extraction Kit (TaKaRa, Dalian) in accordance with the manufacturer’s instructions. First-strand cDNA was synthesized using HiScript II 1st Strand cDNA Synthesis Kit (Vazyme, Nanjing). qRT-PCR was performed according to the procedure of ChamQ Universal SYBR qPCR Master Mix (Vazyme, Nanjing). The Integrated DNA Technologies website (https://sg.idtdna.com/pages) was used to design the primers (Table S1). The relative expression level was normalized to the *GbGAPDH* (GenBank ID: L26924.1) gene and calculated using the 2^−∆∆Ct^ method^73^.

### Statistical analysis

Statistical analyses were performed with IBM SPSS software (Version 22). The relative expression level was presented as the mean ± standard error (SE). Duncan’s multiple range tests were conducted to examine signifcant diferences among means at p < 0.05.

## Supplementary Information

Supplementary Information 1.

Supplementary Information 2.
