# Supplementary material for: Genome-wide characterization of bZIP gene family identifies potential members involved in flavonoids biosynthesis in Ginkgo biloba L
Source: Sci Rep. 2021 Dec 3;11:23420. doi: 10.1038/s41598-021-02839-2 (PMC8642526; doi:10.1038/s41598-021-02839-2)
Supplement: Supplementary file 1 — Supplementary Information 1. [file 41598_2021_2839_MOESM1_ESM.pdf]

Article title: Genome-Wide Characterization of bZIP Gene Family Identifies Potential Members Involved in Flavonoids Biosynthesis in Ginkgo biloba L.

Authors: Huan Han, Feng Xu, Yuting Li, Li Yu, Mingyue Fu, Yongling Liao, Xiaoyan Yang, Weiwei Zhang, Jiabao Ye

Table S1. Primer sequences used in qRT-PCR.

Table S2. Annotations of bZIP protein sequence motifs.

Table S3. Cis-acting element on the promoter of Ginkgo bZIP genes

Table S4. Correlation analysis of flavoniod content and FPKM of 40 GbbZIPs in 8 tissues

Table S5. miRNA family information of GbmiRNAs targeting GbbZIPs

Table S6. flavoniod content in 8 tissues

Table S8. A local blast of *GbbZIP08* in *A. thaliana* and *M. domestica* bZIP protein database

Table S9. A local blast of *GbbZIP15* in *A. thaliana* and *M. domestica* bZIP protein database

Table S1. Primer sequences used in qRT-PCR

Name	Sequence (5'-3')
QbZIP23-U	CCAAACAGACAAGCAGAGAGA
QbZIP23-D	GGATGCCAAAGTAGGGTGAA
QbZIP36-U	TCCATCGGTTCAGCACAATAG
QbZIP36-D	GTAGATGGAAGCAATCCCAGAG
QbZIP35-U	CTTCAGGCAGTACCTGGAAATA
QbZIP35-D	GGATGTCCGTGTTAGGATCAA
QbZIP10-U	ATGAGGCATTGAGGGAAGAAG
QbZIP10-D	GCCTGTTGAGCACCTCTATT
QbZIP33-U	GGTCCCACAACCAACCTAAA
QbZIP33-D	TGAGCAGAAGGAACTGCTATG
QbZIP24-U	TGGCTCTGGCTATTGCTAAC
QbZIP24-D	TGTCGTCCCATACCTCATCTA
QbZIP25-U	GTCGGAAATCCCAGCTTACA
QbZIP25-D	GAATGTCTTTCCAGACCTCCTC
QbZIP15-U	GTCCTGCTGAATCTGGTAGTG
QbZIP15-D	CACGGAGAGCAGGAAAGAAA
QbZIP29-U	ATGGCTTCCAAGGAGGATTG
QbZIP29-D	GTGCCTACACCATCAGAAGAG
QbZIP11-U	CAGTTGCAGGTGGCCTTAT
QbZIP11-D	ATGTGACGGTGGTGTTCTTC
QbZIP16-U	CTTTGGCGGTAGCACCTATAA
QbZIP16-D	GAGGTTGATGGGCTTGTACTT
QbZIP19-U	CCTCCTCTGAGTCCATCTACTT
QbZIP19-D	GGAATTACAGGGCTACCTTCTC
QbZIP40-U	GAAGGGAGTCTTGCACAGTAAT
QbZIP40-D	CTGCTGAAGCCCTGGTTAAT
QbZIP21-U	ATCTCTCCTCAGGTTGCCTAT
QbZIP21-D	TCCTGTACAAGTGCAGCTATTC
QbZIP13-U	CTTGGAGGAGGACTTGCTTTAG
QbZIP13-D	AGGAGATCCAGCTCGAATAGT
QbZIP26-U	TCCGCATCAGGATTCAGTTC
QbZIP26-D	CGTGTTGATCCTCTGCTTCA
QbZIP06-U	TGGACTACTGGAGTGGGTTAG
QbZIP06-D	GTTCTGAGCAGCAGGAATCA
QbZIP08-U	GGATCGAGCATGGAACAGAA
QbZIP08-D	AACGATCACGACACAGAGATG
QbZIP38-U	GCCATTAAACACCTGCAATCC
QbZIP38-D	CCTTCGCAAATGGCAAGAATTA
QbZIP14-U	GAGAACATGGCTCTTAGGACTG
QbZIP14-D	GGTACTGCAACTTCTCTGTGT
GbGAPDH-U	ATCCACGGGAGTCTTCAC
GbGAPDH-D	GACCTTCAACAATGCCAAAC

Table S2. Annotations of bZIP protein sequence motifs

Name	Sequence	Description of Pfam
Motif 1	KRQKRMJSNRESARRSRERKQAYISELERKVQTLKAENSEL	bZIP_1
Motif 2	AQLTLLQRQRVILTNENNALKQRJAALEQQKQLKDALNEAL	NO
Motif 3	TLDEFQNTLGEPGKPFGSMNMDEFLKNIWTAEESQAMAAAM	NO
Motif 4	DVFHLVSGMWKTPAERCFMWMGGFRPSELLKILIPHLEPLTEQQLLGIC	DOG1
Motif 5	TFGEMTLEDFLVKAGVVRED	NO
Motif 6	AGGSATVAGPTTNLNIGMDYWSGSVPAALSSVRGRRAAASTTAAIVPTTQ	MFMR_assoc
Motif 7	SLQRQGSLTLPRTLSRKTVDEVWRDIQ	NO
Motif 8	HACTHTHTCNPPGPDNTHTHTCFHTHTKI	NO
Motif 9	SQEYNQJEEENKVLKSENASLSEKLQKLH	bZIP_1
Motif 10	TLKHQRTPSEGYLQEEQPSWJDDLLDSPSEVVPK	NO
Motif 11	NVANYMGQMAIAMGKLGTLENFVHQADNLRQQTLQQMHRILTTRQAARAL	NO
Motif 12	TTPPPPPGYFPSTVASSPQPHPYMWGAQPLMPPYGTPPPY	MFMR
Motif 13	FDMEYSRWLDEHHRQTNDLRSAVNSHVGDNELRILVDGVMAHYDEIFRLK	DOG1
Motif 14	KHFHSKSMDGSINFNLDFGNGEFSGPELKKIMANEKLAEIAMADP	NO
Motif 15	SHRRSASDSLAFLEAPVSSAPIENIAEE	NO
Motif 16	ZAQQLAQLYQQQQQQ	NO
Motif 17	RDGVPPELWJQDERE	NO
Motif 18	YPDWAAAFQAYY	MFMR
Motif 19	MPDAPPPRKGHRRAHSEIPFRLSDG	NO
Motif 20	TSENPSTPSDHNSINEPSMEEKVMPVSG	NO

Table S3. Cis-acting element on the promoter of *G. biloba* bZIP genes

Gene name	element name	element sequence	position	length	Description
GbbZIP02	G-box	TACGTG	1145	6	light responsive
GbbZIP02	G-box	CACGAC	1685	6	light responsive
GbbZIP02	G-box	CACGTC	1802	6	light responsive
GbbZIP02	TC-rich repeats	GTTTTCTTAC	99	9	defense and stress responsive
GbbZIP02	GT1-motif	GGTTAA	707	6	light responsive
GbbZIP02	ABRE	ACGTG	1146	5	ABA responsive
GbbZIP02	ABRE	ACGTG	1803	5	ABA responsive
GbbZIP02	CGTCA-motif	CGTCA	1385	5	MeJA responsive
GbbZIP02	CGTCA-motif	CGTCA	1628	5	MeJA responsive
GbbZIP02	CGTCA-motif	CGTCA	1801	5	MeJA responsive
GbbZIP02	TGACG-motif	TGACG	1385	5	MeJA responsive
GbbZIP02	TGACG-motif	TGACG	1628	5	MeJA responsive
GbbZIP02	TGACG-motif	TGACG	1801	5	MeJA responsive
GbbZIP23	TCA-element	CCATCTTTTT	1553	9	Salicylic acid responsive
GbbZIP23	TC-rich repeats	ATTCTCTAAC	1382	9	defense and stress responsive
GbbZIP23	MBS	CAACTG	1651	6	MYB binding site
GbbZIP23	ABRE	AACCCGG	1449	7	ABA responsive
GbbZIP23	GARE-motif	TCTGTTG	1000	7	gibberellin responsive
GbbZIP23	GT1-motif	GGTTAA	112	6	light responsive
GbbZIP23	GT1-motif	GGTTAA	126	6	light responsive
GbbZIP23	GT1-motif	GGTTAA	148	6	light responsive
GbbZIP23	GT1-motif	GGTTAA	393	6	light responsive
GbbZIP23	GT1-motif	GGTTAAT	1812	7	light responsive
GbbZIP23	Sp1	GGGCGG	1315	6	light responsive
GbbZIP23	CGTCA-motif	CGTCA	739	5	MeJA responsive
GbbZIP23	ARE	AAACCA	159	6	anoxic inducibility
GbbZIP23	ARE	AAACCA	275	6	anoxic inducibility
GbbZIP23	ARE	AAACCA	1167	6	anoxic inducibility
GbbZIP23	ARE	AAACCA	1573	6	anoxic inducibility
GbbZIP23	MRE	AACCTAA	1823	7	MYB binding site
GbbZIP23	circadian	CAAAGATATC	956	9	circadian control
GbbZIP23	TGACG-motif	TGACG	739	5	MeJA responsive
GbbZIP23	LTR	CCGAAA	920	6	low temperature responsive
GbbZIP30	TGA-element	AACGAC	1016	6	auxin responsive
GbbZIP30	G-box	TACGTG	1189	6	light responsive
GbbZIP30	G-box	CACGTC	1427	6	light responsive
GbbZIP30	G-box	CACGTG	1474	6	light responsive
GbbZIP30	CAT-box	GCCACT	281	6	meristem expression
GbbZIP30	GT1-motif	GGTTAA	1637	6	light responsive
GbbZIP30	ABRE	ACGTG	977	5	ABA responsive
GbbZIP30	ABRE	ACGTG	1190	5	ABA responsive
GbbZIP30	ABRE	ACGTG	1427	5	ABA responsive
GbbZIP30	ABRE	CACGTG	1474	6	ABA responsive

GbbZIP30	ABRE	ACGTG	1475	5	ABA responsive
GbbZIP30	G-Box	CACGTT	976	6	light responsive
GbbZIP30	G-Box	CACGTG	1474	6	light responsive
GbbZIP30	MBS	CAACTG	533	6	MYB binding site
GbbZIP30	MBS	CAACTG	577	6	MYB binding site
GbbZIP30	MBS	CAACTG	707	6	MYB binding site
GbbZIP30	Sp1	GGGCGG	1349	6	light responsive
GbbZIP30	ACE	GACACGTATG	1766	9	light responsive
GbbZIP30	CGTCA-motif	CGTCA	797	5	MeJA responsive
GbbZIP30	CGTCA-motif	CGTCA	1429	5	MeJA responsive
GbbZIP30	TGACG-motif	TGACG	797	5	MeJA responsive
GbbZIP30	TGACG-motif	TGACG	1429	5	MeJA responsive
GbbZIP24	GCN4_motif	TGAGTCA	1819	7	endosperm expression
GbbZIP24	G-Box	TCCACATGGCA	1299	10	light responsive
GbbZIP24	MBS	CAACTG	1482	6	MYB binding site
GbbZIP24	GARE-motif	TCTGTTG	572	7	gibberellin responsive
GbbZIP24	CAT-box	GCCACT	1125	6	meristem expression
GbbZIP24	GT1-motif	GTGTGTGAA	68	9	light responsive
GbbZIP24	GT1-motif	GGTTAAT	588	7	light responsive
GbbZIP24	ABRE	ACGTG	864	5	ABA responsive
GbbZIP24	G-box	TACGTG	863	6	light responsive
GbbZIP24	G-box	GCCACGTGGA	1299	9	light responsive
GbbZIP24	TCA-element	CCATCTTTTT	1522	9	Salicylic acid responsive
GbbZIP24	TCA-element	TCAGAAGAGG	1681	9	Salicylic acid responsive
GbbZIP24	TC-rich repeats	GTTTTCTTAC	353	9	defense and stress responsive
GbbZIP24	TC-rich repeats	GTTTTCTTAC	371	9	defense and stress responsive
GbbZIP24	TC-rich repeats	GTTTTCTTAC	1595	9	defense and stress responsive
GbbZIP24	P-box	CCTTTTG	638	7	gibberellin responsive
GbbZIP24	ACE	CTAACGTATT	16	9	light responsive
GbbZIP27	MRE	AACCTAA	1238	7	MYB binding site
GbbZIP07	ARE	AAACCA	856	6	anoxic inducibility
GbbZIP07	GCN4_motif	TGAGTCA	623	7	endosperm expression
GbbZIP07	CGTCA-motif	CGTCA	1181	5	MeJA responsive
GbbZIP07	LTR	CCGAAA	1388	6	low temperature responsive
GbbZIP07	TGACG-motif	TGACG	1181	5	MeJA responsive
GbbZIP07	GC-motif	CCCCCG	1783	6	anoxic inducibility
GbbZIP07	G-Box	CACGTT	1319	6	light responsive
GbbZIP07	G-Box	CACGTG	1510	6	light responsive
GbbZIP07	ABRE	CGTACGTGCA	1249	9	ABA responsive
GbbZIP07	ABRE	ACGTG	1319	5	ABA responsive
GbbZIP07	ABRE	CGCACGTGTC	1508	9	ABA responsive
GbbZIP07	ABRE	CACGTG	1510	6	ABA responsive
GbbZIP07	ABRE	ACGTG	1511	5	ABA responsive
GbbZIP07	GARE-motif	TCTGTTG	1155	7	gibberellin responsive
GbbZIP07	GARE-motif	TCTGTTG	1865	7	gibberellin responsive

GbbZIP07	CAT-box	GCCACT	166	6	meristem expression
GbbZIP07	CAT-box	GCCACT	1638	6	meristem expression
GbbZIP07	CAT-box	GCCACT	1932	6	meristem expression
GbbZIP07	GT1-motif	GGTTAAT	1102	7	light responsive
GbbZIP07	GT1-motif	GGTTAA	1751	6	light responsive
GbbZIP07	G-box	CACGTG	1510	6	light responsive
GbbZIP07	TC-rich repeats	GTTTTCTTAC	563	9	defense and stress responsive
GbbZIP07	TGA-element	AACGAC	869	6	auxin responsive
GbbZIP07	ACE	GACACGTATG	332	9	light responsive
GbbZIP07	ACE	GCGACGTACC	1459	9	light responsive
GbbZIP07	P-box	CCTTTTG	402	7	gibberellin responsive
GbbZIP07	P-box	CCTTTTG	1843	7	gibberellin responsive
GbbZIP07	Sp1	GGGCGG	1679	6	light responsive
GbbZIP01	ACE	CTAACGTATT	1161	9	light responsive
GbbZIP01	P-box	CCTTTTG	475	7	gibberellin responsive
GbbZIP01	MBS	CAACTG	1008	6	MYB binding site
GbbZIP01	ABRE	ACGTG	795	5	ABA responsive
GbbZIP01	CAT-box	GCCACT	265	6	meristem expression
GbbZIP01	G-box	TACGTG	794	6	light responsive
GbbZIP01	TC-rich repeats	GTTTTCTTAC	1577	9	defense and stress responsive
GbbZIP01	TGACG-motif	TGACG	536	5	MeJA responsive
GbbZIP01	ARE	AAACCA	1144	6	anoxic inducibility
GbbZIP01	ARE	AAACCA	1228	6	anoxic inducibility
GbbZIP01	ARE	AAACCA	1476	6	anoxic inducibility
GbbZIP01	MRE	AACCTAA	865	7	MYB binding site
GbbZIP01	MRE	AACCTAA	1416	7	MYB binding site
GbbZIP01	CGTCA-motif	CGTCA	536	5	MeJA responsive
GbbZIP03	ARE	AAACCA	189	6	anoxic inducibility
GbbZIP03	CGTCA-motif	CGTCA	1664	5	MeJA responsive
GbbZIP03	TGACG-motif	TGACG	1664	5	MeJA responsive
GbbZIP03	TGA-element	AACGAC	1332	6	auxin responsive
GbbZIP03	TC-rich repeats	GTTTTCTTAC	49	10	defense and stress responsive
GbbZIP03	TCA-element	CCATCTTTTT	1364	9	Salicylic acid responsive
GbbZIP36	RY-element	CATGCATG	1303	8	seed-specific regulation
GbbZIP36	TGACG-motif	TGACG	94	5	MeJA responsive
GbbZIP36	ARE	AAACCA	746	6	anoxic inducibility
GbbZIP36	ARE	AAACCA	1794	6	anoxic inducibility
GbbZIP36	CGTCA-motif	CGTCA	94	5	MeJA responsive
GbbZIP36	P-box	CCTTTTG	49	7	gibberellin responsive
GbbZIP36	P-box	CCTTTTG	657	7	gibberellin responsive
GbbZIP36	MBS	CAACTG	1798	6	MYB binding site
GbbZIP36	MBS	CAACTG	1873	6	MYB binding site
GbbZIP36	GT1-motif	GGTTAAT	1229	7	light responsive
GbbZIP36	GT1-motif	GGTTAA	1230	6	light responsive
GbbZIP36	ABRE	AACCCGG	282	7	ABA responsive

GbbZIP36	TCA-element	CCATCTTTTT	240	9	Salicylic acid responsive
GbbZIP36	TCA-element	TCAGAAGAGG	1662	9	Salicylic acid responsive
GbbZIP36	TC-rich repeats	GTTTTCTTAC	1915	9	defense and stress responsive
GbbZIP25	P-box	CCTTTTG	1748	7	gibberellin responsive
GbbZIP25	G-box	TAACACGTAG	811	9	light responsive
GbbZIP25	G-box	TACGTG	814	6	light responsive
GbbZIP25	G-box	CACGAC	1775	6	light responsive
GbbZIP25	G-box	CACGTC	1838	6	light responsive
GbbZIP25	TCA-element	CCATCTTTTT	1665	9	Salicylic acid responsive
GbbZIP25	TGA-element	AACGAC	1621	6	auxin responsive
GbbZIP25	TC-rich repeats	GTTTTCTTAC	1422	9	defense and stress responsive
GbbZIP25	GT1-motif	GGTTAA	24	6	light responsive
GbbZIP25	GT1-motif	GGTTAAT	181	7	light responsive
GbbZIP25	GT1-motif	GGTTAA	182	6	light responsive
GbbZIP25	ABRE	ACGTG	814	5	ABA responsive
GbbZIP25	ABRE	AACCCGG	1014	7	ABA responsive
GbbZIP25	ABRE	ACGTG	1838	5	ABA responsive
GbbZIP25	GC-motif	CCCCCG	1159	6	anoxic inducibility
GbbZIP25	ARE	AAACCA	541	6	anoxic inducibility
GbbZIP25	ARE	AAACCA	583	6	anoxic inducibility
GbbZIP15	ARE	AAACCA	1349	6	anoxic inducibility
GbbZIP15	ARE	AAACCA	1516	6	anoxic inducibility
GbbZIP15	circadian	CAAAGATATC	1743	9	circadian control
GbbZIP15	AuxRR-core	GGTCCAT	757	7	auxin responsive
GbbZIP15	G-box	CACGTC	542	6	light responsive
GbbZIP15	G-box	CACGTG	552	6	light responsive
GbbZIP15	G-box	CACGAC	1611	6	light responsive
GbbZIP15	G-box	CACGAC	1972	6	light responsive
GbbZIP15	TC-rich repeats	GTTTTCTTAC	1760	9	defense and stress responsive
GbbZIP15	TGA-element	AACGAC	1501	6	auxin responsive
GbbZIP15	G-Box	CACGTG	552	6	light responsive
GbbZIP15	G-Box	CACGTT	1633	6	light responsive
GbbZIP15	ABRE	ACGTG	543	5	ABA responsive
GbbZIP15	ABRE	CACGTG	552	6	ABA responsive
GbbZIP15	ABRE	ACGTG	553	5	ABA responsive
GbbZIP15	ABRE	ACGTG	1633	5	ABA responsive
GbbZIP15	ACE	CTAACGTATT	729	9	light responsive
GbbZIP15	P-box	CCTTTTG	822	7	gibberellin responsive
GbbZIP15	P-box	CCTTTTG	1795	7	gibberellin responsive
GbbZIP16	TGACG-motif	TGACG	735	5	MeJA responsive
GbbZIP16	TGACG-motif	TGACG	804	5	MeJA responsive
GbbZIP16	TGACG-motif	TGACG	1159	5	MeJA responsive
GbbZIP16	ARE	AAACCA	483	6	anoxic inducibility
GbbZIP16	ARE	AAACCA	571	6	anoxic inducibility
GbbZIP16	ARE	AAACCA	682	6	anoxic inducibility

GbbZIP16	ARE	AAACCA	1822	6	anoxic inducibility
GbbZIP16	CGTCA-motif	CGTCA	735	5	MeJA responsive
GbbZIP16	CGTCA-motif	CGTCA	804	5	MeJA responsive
GbbZIP16	CGTCA-motif	CGTCA	1159	5	MeJA responsive
GbbZIP16	G-Box	CACGTT	745	6	light responsive
GbbZIP16	MBS	CAACTG	908	6	MYB binding site
GbbZIP16	MBS	CAACTG	1231	6	MYB binding site
GbbZIP16	CAT-box	GCCACT	1316	6	meristem expression
GbbZIP16	ABRE	GCAACGTGTC	743	10	ABA responsive
GbbZIP16	ABRE	ACGTG	746	5	ABA responsive
GbbZIP16	ABRE	ACGTG	802	5	ABA responsive
GbbZIP16	ABRE	GACACGTGGC	1358	9	ABA responsive
GbbZIP16	G-box	CACGAC	390	6	light responsive
GbbZIP16	G-box	CACGTC	802	6	light responsive
GbbZIP16	G-box	tgACACGTGGCA	1357	11	light responsive
GbbZIP16	TCA-element	TCAGAAGAGG	98	10	Salicylic acid responsive
GbbZIP16	TGA-element	AACGAC	762	6	auxin responsive
GbbZIP37	RY-element	CATGCATG	1791	8	seed-specific regulation
GbbZIP37	MRE	AACCTAA	569	7	MYB binding site
GbbZIP37	ARE	AAACCA	895	6	anoxic inducibility
GbbZIP37	circadian	CAAAGATATC	1154	9	circadian control
GbbZIP37	P-box	CCTTTTG	1467	7	gibberellin responsive
GbbZIP37	MBS	CAACTG	1231	6	MYB binding site
GbbZIP37	CAT-box	GCCACT	495	6	meristem expression
GbbZIP37	GT1-motif	GGTTAA	572	6	light responsive
GbbZIP37	GT1-motif	GGTTAA	1096	6	light responsive
GbbZIP37	ABRE	ACGTG	304	5	ABA responsive
GbbZIP37	G-box	CACGTC	303	6	light responsive
GbbZIP37	AuxRR-core	GGTCCAT	150	7	auxin responsive
GbbZIP37	AuxRR-core	GGTCCAT	1274	7	auxin responsive
GbbZIP35	TGACG-motif	TGACG	226	5	MeJA responsive
GbbZIP35	TGACG-motif	TGACG	467	5	MeJA responsive
GbbZIP35	TGACG-motif	TGACG	1006	5	MeJA responsive
GbbZIP35	TGACG-motif	TGACG	1368	5	MeJA responsive
GbbZIP35	RY-element	CATGCATG	116	8	seed-specific regulation
GbbZIP35	LTR	CCGAAA	419	6	low temperature responsive
GbbZIP35	LTR	CCGAAA	566	6	low temperature responsive
GbbZIP35	LTR	CCGAAA	574	6	low temperature responsive
GbbZIP35	LTR	CCGAAA	807	6	low temperature responsive
GbbZIP35	CGTCA-motif	CGTCA	226	5	MeJA responsive
GbbZIP35	CGTCA-motif	CGTCA	467	5	MeJA responsive
GbbZIP35	CGTCA-motif	CGTCA	1006	5	MeJA responsive
GbbZIP35	CGTCA-motif	CGTCA	1368	5	MeJA responsive
GbbZIP35	ARE	AAACCA	1055	6	anoxic inducibility
GbbZIP35	ARE	AAACCA	1124	6	anoxic inducibility

GbbZIP35	ARE	AAACCA	1151	6	anoxic inducibility
GbbZIP35	ARE	AAACCA	1281	6	anoxic inducibility
GbbZIP35	ARE	AAACCA	1941	6	anoxic inducibility
GbbZIP35	Sp1	GGGCGG	83	6	light responsive
GbbZIP35	Sp1	GGGCGG	451	6	light responsive
GbbZIP35	Sp1	GGGCGG	671	6	light responsive
GbbZIP35	P-box	CCTTTTG	78	7	gibberellin responsive
GbbZIP35	P-box	CCTTTTG	1762	7	gibberellin responsive
GbbZIP35	TGA-element	AACGAC	510	6	auxin responsive
GbbZIP35	TGA-element	AACGAC	589	6	auxin responsive
GbbZIP35	AuxRR-core	GGTCCAT	67	7	auxin responsive
GbbZIP35	G-box	CACGAC	218	6	light responsive
GbbZIP35	G-box	GCCACGTGGA	1307	9	light responsive
GbbZIP35	TCA-element	CCATCTTTTT	147	9	Salicylic acid responsive
GbbZIP35	ABRE	ACGTG	1924	5	ABA responsive
GbbZIP35	GT1-motif	GGTTAA	1708	6	light responsive
GbbZIP35	G-Box	CACGTT	1924	6	light responsive
GbbZIP29	LTR	CCGAAA	102	6	low temperature responsive
GbbZIP29	TGACG-motif	TGACG	206	5	MeJA responsive
GbbZIP29	GC-motif	CCCCCG	376	6	anoxic inducibility
GbbZIP29	GC-motif	CCCCCG	510	6	anoxic inducibility
GbbZIP29	circadian	CAAAGATATC	338	9	circadian control
GbbZIP29	ARE	AAACCA	1072	6	anoxic inducibility
GbbZIP29	ARE	AAACCA	1657	6	anoxic inducibility
GbbZIP29	CGTCA-motif	CGTCA	206	5	MeJA responsive
GbbZIP29	ABRE	ACGTG	166	5	ABA responsive
GbbZIP29	ABRE	ACGTG	175	5	ABA responsive
GbbZIP29	ABRE	CACGTG	238	6	ABA responsive
GbbZIP29	ABRE	ACGTG	239	5	ABA responsive
GbbZIP29	ABRE	CGCACGTGTC	288	9	ABA responsive
GbbZIP29	ABRE	CACGTG	290	6	ABA responsive
GbbZIP29	ABRE	ACGTG	291	5	ABA responsive
GbbZIP29	ABRE	GCCGCGTGGC	501	9	ABA responsive
GbbZIP29	GT1-motif	GGTTAAT	629	7	light responsive
GbbZIP29	GT1-motif	GGTTAA	630	6	light responsive
GbbZIP29	G-Box	CACGTT	175	6	light responsive
GbbZIP29	G-Box	CACGTG	238	6	light responsive
GbbZIP29	G-Box	CACGTG	290	6	light responsive
GbbZIP29	TC-rich repeats	GTTTTCTTAC	268	9	defense and stress responsive
GbbZIP29	TGA-element	AACGAC	866	6	auxin responsive
GbbZIP29	TGA-element	AACGAC	1439	6	auxin responsive
GbbZIP29	AuxRR-core	GGTCCAT	1143	7	auxin responsive
GbbZIP29	G-box	TACGTG	166	6	light responsive
GbbZIP29	G-box	CACGTG	238	6	light responsive
GbbZIP29	G-box	CACGTG	290	6	light responsive

GbbZIP13	GC-motif	CCCCCG	1011	6	anoxic inducibility
GbbZIP13	TATC-box	TATCCCA	2	7	gibberellin responsive
GbbZIP13	ACE	GACACGTATG	636	9	light responsive
GbbZIP13	ACE	GACACGTATG	698	9	light responsive
GbbZIP13	TCA-element	CCATCTTTTT	727	9	Salicylic acid responsive
GbbZIP13	TCA-element	CCATCTTTTT	1746	9	Salicylic acid responsive
GbbZIP13	G-box	TACGTG	638	6	light responsive
GbbZIP13	G-box	ACACGTGT	699	8	light responsive
GbbZIP13	G-box	CACGTG	700	6	light responsive
GbbZIP13	G-box	CACGAC	771	6	light responsive
GbbZIP13	G-box	ACACGTG(G/t)CACC	1076	10.5	light responsive
GbbZIP13	ABRE	TACGTGTC	636	8	ABA responsive
GbbZIP13	ABRE	ACGTG	638	5	ABA responsive
GbbZIP13	ABRE	CGCACGTGTC	698	9	ABA responsive
GbbZIP13	ABRE	CACGTG	700	6	ABA responsive
GbbZIP13	ABRE	ACGTG	701	5	ABA responsive
GbbZIP13	ABRE	AACCCGG	1007	7	ABA responsive
GbbZIP13	G-Box	CACGTG	700	6	light responsive
GbbZIP19	LTR	CCGAAA	1350	6	low temperature responsive
GbbZIP19	SARE	TTCGACCATCTT	1386	11	Salicylic acid responsive
GbbZIP19	TGACG-motif	TGACG	133	5	MeJA responsive
GbbZIP19	TGACG-motif	TGACG	1266	5	MeJA responsive
GbbZIP19	TGACG-motif	TGACG	1601	5	MeJA responsive
GbbZIP19	TGACG-motif	TGACG	1682	5	MeJA responsive
GbbZIP19	ARE	AAACCA	1851	6	anoxic inducibility
GbbZIP19	ARE	AAACCA	1962	6	anoxic inducibility
GbbZIP19	CGTCA-motif	CGTCA	133	5	MeJA responsive
GbbZIP19	CGTCA-motif	CGTCA	1266	5	MeJA responsive
GbbZIP19	CGTCA-motif	CGTCA	1601	5	MeJA responsive
GbbZIP19	CGTCA-motif	CGTCA	1682	5	MeJA responsive
GbbZIP19	G-Box	CACGTT	1019	6	light responsive
GbbZIP19	G-Box	CACGTG	1143	6	light responsive
GbbZIP19	CAT-box	GCCACT	1182	6	meristem expression
GbbZIP19	GT1-motif	GGTTAA	730	6	light responsive
GbbZIP19	ABRE	ACGTG	131	5	ABA responsive
GbbZIP19	ABRE	ACGTG	1020	5	ABA responsive
GbbZIP19	ABRE	CACGTG	1143	6	ABA responsive
GbbZIP19	ABRE	ACGTG	1144	5	ABA responsive
GbbZIP19	G-box	CACGTC	131	6	light responsive
GbbZIP19	G-box	GCCACGTGGA	1141	10	light responsive
GbbZIP19	G-box	CACGTG	1143	6	light responsive
GbbZIP26	P-box	CCTTTTG	1225	7	gibberellin responsive
GbbZIP26	P-box	CCTTTTG	1601	7	gibberellin responsive
GbbZIP26	3-AF1 binding site	TAAGAGAGGAA	23	10	light responsive
GbbZIP26	TCA-element	CCATCTTTTT	1316	9	Salicylic acid responsive

GbbZIP26	TGA-element	AACGAC	1562	6	auxin responsive
GbbZIP26	MBS	CAACTG	1532	6	MYB binding site
GbbZIP26	G-Box	TCCACATGGCA	1539	10	light responsive
GbbZIP26	GARE-motif	TCTGTTG	1580	7	gibberellin responsive
GbbZIP26	CAT-box	GCCACT	1338	6	meristem expression
GbbZIP26	TGACG-motif	TGACG	145	5	MeJA responsive
GbbZIP26	CGTCA-motif	CGTCA	145	5	MeJA responsive
GbbZIP26	GCN4_motif	TGAGTCA	160	7	endosperm expression
GbbZIP26	GCN4_motif	TGAGTCA	1347	7	endosperm expression
GbbZIP26	ARE	AAACCA	19	6	anoxic inducibility
GbbZIP26	ARE	AAACCA	958	6	anoxic inducibility
GbbZIP26	ARE	AAACCA	1447	6	anoxic inducibility
GbbZIP32	ARE	AAACCA	14	6	anoxic inducibility
GbbZIP32	ARE	AAACCA	174	6	anoxic inducibility
GbbZIP32	MBSI	aaaAaaC(G/C)GTTA	335	10.5	MYB binding site
GbbZIP32	circadian	CAAAGATATC	29	9	circadian control
GbbZIP32	GCN4_motif	TGAGTCA	1172	7	endosperm expression
GbbZIP32	MBS	CAACTG	782	6	MYB binding site
GbbZIP32	MBS	CAACTG	1463	6	MYB binding site
GbbZIP32	MBS	CAACTG	1708	6	MYB binding site
GbbZIP32	GT1-motif	GGTTAAT	815	7	light responsive
GbbZIP32	GT1-motif	GGTTAA	816	6	light responsive
GbbZIP32	CAT-box	GCCACT	317	6	meristem expression
GbbZIP32	CAT-box	GCCACT	892	6	meristem expression
GbbZIP32	CAT-box	GCCACT	1163	6	meristem expression
GbbZIP32	TCA-element	TCAGAAGAGG	1373	9	Salicylic acid responsive
GbbZIP39	ARE	AAACCA	768	6	anoxic inducibility
GbbZIP39	CGTCA-motif	CGTCA	150	5	MeJA responsive
GbbZIP39	CGTCA-motif	CGTCA	1308	5	MeJA responsive
GbbZIP39	TGACG-motif	TGACG	150	5	MeJA responsive
GbbZIP39	TGACG-motif	TGACG	1308	5	MeJA responsive
GbbZIP39	ABRE	ACGTG	1832	5	ABA responsive
GbbZIP39	TC-rich repeats	ATTCTCTAAC	1047	9	defense and stress responsive
GbbZIP39	G-box	CACGAC	240	6	light responsive
GbbZIP39	G-box	CACGAC	1075	6	light responsive
GbbZIP39	G-box	tcCACGTGTCACT	1156	12	light responsive
GbbZIP39	G-box	TACGTG	1832	6	light responsive
GbbZIP39	ACE	CTAACGTATT	646	9	light responsive
GbbZIP06	G-Box	TCCACATGGCA	378	10	light responsive
GbbZIP06	CAT-box	GCCACT	499	6	meristem expression
GbbZIP06	CAT-box	GCCACT	1447	6	meristem expression
GbbZIP06	GARE-motif	TCTGTTG	789	7	gibberellin responsive
GbbZIP06	G-box	CACGAC	215	6	light responsive
GbbZIP06	G-box	CACGAC	431	6	light responsive
GbbZIP06	TCA-element	TCAGAAGAGG	405	9	Salicylic acid responsive

GbbZIP06	TGA-element	AACGAC	1349	6	auxin responsive
GbbZIP06	P-box	CCTTTTG	175	7	gibberellin responsive
GbbZIP06	ARE	AAACCA	367	6	anoxic inducibility
GbbZIP06	ARE	AAACCA	980	6	anoxic inducibility
GbbZIP40	G-box	CACGAC	849	6	light responsive
GbbZIP40	G-box	TACGTG	1849	6	light responsive
GbbZIP40	G-box	TAAACGTG	1866	8	light responsive
GbbZIP40	TCA-element	CCATCTTTTT	1968	9	Salicylic acid responsive
GbbZIP40	G-Box	CACGTT	1786	6	light responsive
GbbZIP40	G-Box	CACGTT	1866	6	light responsive
GbbZIP40	MBS	CAACTG	1706	6	MYB binding site
GbbZIP40	ABRE	ACGTG	1787	5	ABA responsive
GbbZIP40	ABRE	ACGTG	1850	5	ABA responsive
GbbZIP40	ABRE	ACGTG	1866	5	ABA responsive
GbbZIP40	GT1-motif	GGTTAA	1832	6	light responsive
GbbZIP40	Sp1	GGGCGG	420	6	light responsive
GbbZIP40	CGTCA-motif	CGTCA	1031	5	MeJA responsive
GbbZIP40	ARE	AAACCA	69	6	anoxic inducibility
GbbZIP40	ARE	AAACCA	1295	6	anoxic inducibility
GbbZIP40	ARE	AAACCA	1937	6	anoxic inducibility
GbbZIP40	TGACG-motif	TGACG	1031	5	MeJA responsive
GbbZIP34	LTR	CCGAAA	298	6	low temperature responsive
GbbZIP34	ARE	AAACCA	1283	6	anoxic inducibility
GbbZIP34	ARE	AAACCA	1961	6	anoxic inducibility
GbbZIP34	circadian	CAAAGATATC	1295	9	circadian control
GbbZIP34	GCN4_motif	TGAGTCA	569	7	endosperm expression
GbbZIP34	MBS	CAACTG	562	6	MYB binding site
GbbZIP34	MBS	CAACTG	847	6	MYB binding site
GbbZIP34	CAT-box	GCCACT	809	6	meristem expression
GbbZIP34	GARE-motif	TCTGTTG	1986	7	gibberellin responsive
GbbZIP34	TCA-element	CCATCTTTTT	1811	9	Salicylic acid responsive
GbbZIP21	CGTCA-motif	CGTCA	1001	5	MeJA responsive
GbbZIP21	circadian	CAAAGATATC	1741	9	circadian control
GbbZIP21	ARE	AAACCA	227	6	anoxic inducibility
GbbZIP21	ARE	AAACCA	731	6	anoxic inducibility
GbbZIP21	ARE	AAACCA	924	6	anoxic inducibility
GbbZIP21	ARE	AAACCA	1934	6	anoxic inducibility
GbbZIP21	TGACG-motif	TGACG	1001	5	MeJA responsive
GbbZIP21	LTR	CCGAAA	1183	6	low temperature responsive
GbbZIP21	TC-rich repeats	GTTTTCTTAC	1020	9	defense and stress responsive
GbbZIP21	TGA-element	AACGAC	1422	6	auxin responsive
GbbZIP21	G-box	GCCACGTGGA	328	9	light responsive
GbbZIP21	G-box	CACGTG	330	6	light responsive
GbbZIP21	G-box	TACGTG	404	6	light responsive
GbbZIP21	TCA-element	CCATCTTTTT	591	9	Salicylic acid responsive

GbbZIP21	ABRE	CACGTG	330	6	ABA responsive
GbbZIP21	ABRE	ACGTG	331	5	ABA responsive
GbbZIP21	ABRE	GCAACGTGTC	402	9	ABA responsive
GbbZIP21	ABRE	TACGTGTC	404	8	ABA responsive
GbbZIP21	ABRE	ACGTG	405	5	ABA responsive
GbbZIP21	CAT-box	GCCACT	1852	6	meristem expression
GbbZIP21	MBS	CAACTG	199	6	MYB binding site
GbbZIP21	G-Box	CACGTG	330	6	light responsive
GbbZIP08	ARE	AAACCA	1311	6	anoxic inducibility
GbbZIP08	G-box	TACGTG	1487	6	light responsive
GbbZIP08	G-box	TACGTG	1891	6	light responsive
GbbZIP08	G-box	CACGTG	1942	6	light responsive
GbbZIP08	TCA-element	CCATCTTTTT	398	9	Salicylic acid responsive
GbbZIP08	CAT-box	GCCACT	1218	6	meristem expression
GbbZIP08	CAT-box	GCCACT	1482	6	meristem expression
GbbZIP08	GT1-motif	GGTTAAT	229	7	light responsive
GbbZIP08	GT1-motif	GGTTAA	230	6	light responsive
GbbZIP08	GT1-motif	GGTTAAT	803	7	light responsive
GbbZIP08	GT1-motif	GGTTAA	804	6	light responsive
GbbZIP08	ABRE	CGCACGTGTC	329	9	ABA responsive
GbbZIP08	ABRE	ACGTG	331	5	ABA responsive
GbbZIP08	ABRE	ACGTG	1488	5	ABA responsive
GbbZIP08	ABRE	ACGTG	1892	5	ABA responsive
GbbZIP08	ABRE	CACGTG	1942	6	ABA responsive
GbbZIP08	ABRE	ACGTG	1943	5	ABA responsive
GbbZIP08	G-Box	CACGTT	331	6	light responsive
GbbZIP08	G-Box	CACGTG	1942	6	light responsive
GbbZIP08	3-AF1 binding site	TAAGAGAGGAA	62	10	light responsive
GbbZIP22	TGACG-motif	TGACG	978	5	MeJA responsive
GbbZIP22	TGACG-motif	TGACG	1727	5	MeJA responsive
GbbZIP22	RY-element	CATGCATG	781	8	seed-specific regulation
GbbZIP22	CGTCA-motif	CGTCA	978	5	MeJA responsive
GbbZIP22	CGTCA-motif	CGTCA	1727	5	MeJA responsive
GbbZIP22	ARE	AAACCA	417	6	anoxic inducibility
GbbZIP22	circadian	CAAAGATATC	1256	9	circadian control
GbbZIP22	P-box	CCTTTTG	1830	7	gibberellin responsive
GbbZIP22	TGA-element	AACGAC	233	6	auxin responsive
GbbZIP22	TC-rich repeats	GTTTTCTTAC	1065	9	defense and stress responsive
GbbZIP22	TC-rich repeats	GTTTTCTTAC	1085	9	defense and stress responsive
GbbZIP22	MBS	CAACTG	71	6	MYB binding site
GbbZIP22	CAT-box	GCCACT	1904	6	meristem expression
GbbZIP22	GT1-motif	GGTTAA	145	6	light responsive
GbbZIP22	GT1-motif	GGTTAAT	245	7	light responsive
GbbZIP22	GT1-motif	GGTTAA	527	6	light responsive
GbbZIP11	TGACG-motif	TGACG	1003	5	MeJA responsive

GbbZIP11	TGACG-motif	TGACG	1632	5	MeJA responsive
GbbZIP11	TGACG-motif	TGACG	1734	5	MeJA responsive
GbbZIP11	CGTCA-motif	CGTCA	1003	5	MeJA responsive
GbbZIP11	CGTCA-motif	CGTCA	1632	5	MeJA responsive
GbbZIP11	CGTCA-motif	CGTCA	1734	5	MeJA responsive
GbbZIP11	ARE	AAACCA	224	6	anoxic inducibility
GbbZIP11	ARE	AAACCA	1769	6	anoxic inducibility
GbbZIP11	G-box	CACGAC	1363	6	light responsive
GbbZIP11	TGA-element	AACGAC	1798	6	auxin responsive
GbbZIP11	ABRE	GACACGTACGT	965	10	ABA responsive
GbbZIP11	CAT-box	GCCACT	341	6	meristem expression
GbbZIP11	CAT-box	GCCACT	1399	6	meristem expression
GbbZIP11	GARE-motif	TCTGTTG	998	7	gibberellin responsive
GbbZIP11	GARE-motif	TCTGTTG	1506	7	gibberellin responsive
GbbZIP12	ABRE	ACGTG	231	5	ABA responsive
GbbZIP12	ABRE	TACGTGTC	342	8	ABA responsive
GbbZIP12	ABRE	ACGTG	343	5	ABA responsive
GbbZIP12	ABRE	TACGGTC	1088	7	ABA responsive
GbbZIP12	ABRE	TACGGTC	1330	7	ABA responsive
GbbZIP12	ABRE	GCAACGTGTC	1688	9	ABA responsive
GbbZIP12	TGA-element	AACGAC	1647	6	auxin responsive
GbbZIP12	G-box	CACGAC	129	6	light responsive
GbbZIP12	G-box	CACGTC	231	6	light responsive
GbbZIP12	G-box	TACGTG	342	6	light responsive
GbbZIP12	ARE	AAACCA	1251	6	anoxic inducibility
GbbZIP12	ARE	AAACCA	1293	6	anoxic inducibility
GbbZIP12	MRE	AACCTAA	121	7	MYB binding site
GbbZIP12	GCN4_motif	TGAGTCA	185	7	endosperm expression
GbbZIP05	P-box	CCTTTTG	1100	7	gibberellin responsive
GbbZIP05	Sp1	GGGCGG	1904	6	light responsive
GbbZIP05	TGA-element	AACGAC	1750	6	auxin responsive
GbbZIP05	MBS	CAACTG	1649	6	MYB binding site
GbbZIP05	GARE-motif	TCTGTTG	1874	7	gibberellin responsive
GbbZIP05	GT1-motif	GGTTAA	1200	6	light responsive
GbbZIP05	ABRE	TACGGTC	1563	7	ABA responsive
GbbZIP05	LTR	CCGAAA	1815	6	low temperature responsive
GbbZIP05	RY-element	CATGCATG	584	8	seed-specific regulation
GbbZIP05	ARE	AAACCA	1633	6	anoxic inducibility
GbbZIP09	CGTCA-motif	CGTCA	676	5	MeJA responsive
GbbZIP09	TGACG-motif	TGACG	676	5	MeJA responsive
GbbZIP09	TC-rich repeats	GTTTTCTTAC	476	9	defense and stress responsive
GbbZIP09	TC-rich repeats	ATTCTCTAAC	1470	9	defense and stress responsive
GbbZIP09	TCA-element	CCATCTTTTT	213	9	Salicylic acid responsive
GbbZIP09	G-Box	TCCACATGGCA	380	10	light responsive
GbbZIP28	ARE	AAACCA	613	6	anoxic inducibility

GbbZIP28	ARE	AAACCA	1239	6	anoxic inducibility
GbbZIP28	ARE	AAACCA	1581	6	anoxic inducibility
GbbZIP28	GCN4_motif	TGAGTCA	1257	7	endosperm expression
GbbZIP28	G-box	CACGAC	1020	6	light responsive
GbbZIP28	TCA-element	CCATCTTTTT	1203	9	Salicylic acid responsive
GbbZIP28	TC-rich repeats	GTTTTCTTAC	563	9	defense and stress responsive
GbbZIP28	TGA-element	AACGAC	1092	6	auxin responsive
GbbZIP04	ARE	AAACCA	1847	6	anoxic inducibility
GbbZIP04	ARE	AAACCA	1852	6	anoxic inducibility
GbbZIP04	TATC-box	TATCCCA	1246	7	gibberellin responsive
GbbZIP04	MBS	CAACTG	101	6	MYB binding site
GbbZIP04	MBS	CAACTG	398	6	MYB binding site
GbbZIP04	MBS	CAACTG	797	6	MYB binding site
GbbZIP04	MBS	CAACTG	1138	6	MYB binding site
GbbZIP04	GT1-motif	GGTTAA	1976	6	light responsive
GbbZIP04	TC-rich repeats	GTTTTCTTAC	1478	9	defense and stress responsive
GbbZIP04	P-box	CCTTTTG	824	7	gibberellin responsive
GbbZIP20	MBS	CAACTG	1888	6	MYB binding site
GbbZIP20	CAT-box	GCCACT	1697	6	meristem expression
GbbZIP20	G-box	CACGAC	862	6	light responsive
GbbZIP20	TGA-element	AACGAC	1494	6	auxin responsive
GbbZIP20	TGA-element	AACGAC	1588	6	auxin responsive
GbbZIP20	ACE	CTAACGTATT	227	9	light responsive
GbbZIP20	MRE	AACCTAA	788	7	MYB binding site
GbbZIP20	ARE	AAACCA	1097	6	anoxic inducibility
GbbZIP20	ARE	AAACCA	1311	6	anoxic inducibility
GbbZIP20	circadian	CAAAGATATC	750	9	circadian control
GbbZIP20	circadian	CAAAGATATC	754	9	circadian control
GbbZIP20	GCN4_motif	TGAGTCA	125	7	endosperm expression
GbbZIP20	LTR	CCGAAA	724	6	low temperature responsive
GbbZIP20	LTR	CCGAAA	1202	6	low temperature responsive
GbbZIP38	TGACG-motif	TGACG	263	5	MeJA responsive
GbbZIP38	TGACG-motif	TGACG	830	5	MeJA responsive
GbbZIP38	CGTCA-motif	CGTCA	263	5	MeJA responsive
GbbZIP38	CGTCA-motif	CGTCA	830	5	MeJA responsive
GbbZIP38	circadian	CAAAGATATC	403	9	circadian control
GbbZIP38	ARE	AAACCA	947	6	anoxic inducibility
GbbZIP38	MRE	AACCTAA	451	7	MYB binding site
GbbZIP38	MRE	AACCTAA	1082	7	MYB binding site
GbbZIP38	TC-rich repeats	GTTTTCTTAC	1429	9	defense and stress responsive
GbbZIP38	TC-rich repeats	GTTTTCTTAC	1771	9	defense and stress responsive
GbbZIP38	TC-rich repeats	GTTTTCTTAC	1845	9	defense and stress responsive
GbbZIP38	TCA-element	CCATCTTTTT	1262	9	Salicylic acid responsive
GbbZIP38	G-box	TACGTG	795	6	light responsive
GbbZIP38	G-box	TACGTG	1315	6	light responsive

GbbZIP38	ABRE	ACGTG	795	5	ABA responsive
GbbZIP38	ABRE	ACGTG	1316	5	ABA responsive
GbbZIP38	GT1-motif	GGTTAAT	387	7	light responsive
GbbZIP38	GT1-motif	GGTTAA	1354	6	light responsive
GbbZIP38	CAT-box	GCCACT	44	6	meristem expression
GbbZIP38	CAT-box	GCCACT	701	6	meristem expression
GbbZIP17	TGACG-motif	TGACG	1006	5	MeJA responsive
GbbZIP17	WUN-motif	AAATTTCCT	1803	9	wound responsive
GbbZIP17	GCN4_motif	TGAGTCA	428	7	endosperm expression
GbbZIP17	CGTCA-motif	CGTCA	1006	5	MeJA responsive
GbbZIP17	ARE	AAACCA	456	6	anoxic inducibility
GbbZIP17	ARE	AAACCA	496	6	anoxic inducibility
GbbZIP17	ARE	AAACCA	549	6	anoxic inducibility
GbbZIP17	ARE	AAACCA	950	6	anoxic inducibility
GbbZIP17	Sp1	GGGCGG	1013	6	light responsive
GbbZIP17	TC-rich repeats	ATTCTCTAAC	336	9	defense and stress responsive
GbbZIP17	TC-rich repeats	GTTTTCTTAC	1428	9	defense and stress responsive
GbbZIP17	TGA-element	AACGAC	1845	6	auxin responsive
GbbZIP17	G-Box	CACGTT	1457	6	light responsive
GbbZIP17	MBS	CAACTG	610	6	MYB binding site
GbbZIP17	MBS	CAACTG	736	6	MYB binding site
GbbZIP17	MBS	CAACTG	1634	6	MYB binding site
GbbZIP17	MBS	CAACTG	1721	6	MYB binding site
GbbZIP17	MBS	CAACTG	1870	6	MYB binding site
GbbZIP17	ABRE	GCAACGTGTC	1175	9	ABA responsive
GbbZIP17	ABRE	ACGTG	1458	5	ABA responsive
GbbZIP17	GT1-motif	GGTTAA	284	6	light responsive
GbbZIP14	AuxRR-core	GGTCCAT	251	7	auxin responsive
GbbZIP14	TCA-element	TCAGAAGAGG	278	9	Salicylic acid responsive
GbbZIP14	TCA-element	TCAGAAGAGG	1343	9	Salicylic acid responsive
GbbZIP14	ABRE	TACGGTC	1280	7	ABA responsive
GbbZIP14	CAT-box	GCCACT	112	6	meristem expression
GbbZIP14	CAT-box	GCCACT	579	6	meristem expression
GbbZIP14	GARE-motif	TCTGTTG	1314	7	gibberellin responsive
GbbZIP14	MBS	CAACTG	753	6	MYB binding site
GbbZIP14	MBS	CAACTG	1635	6	MYB binding site
GbbZIP14	GCN4_motif	TGAGTCA	104	7	endosperm expression
GbbZIP14	ARE	AAACCA	345	6	anoxic inducibility
GbbZIP14	ARE	AAACCA	552	6	anoxic inducibility
GbbZIP14	ARE	AAACCA	814	6	anoxic inducibility
GbbZIP14	ARE	AAACCA	1232	6	anoxic inducibility
GbbZIP14	ARE	AAACCA	1402	6	anoxic inducibility
GbbZIP10	ARE	AAACCA	3	6	anoxic inducibility
GbbZIP10	ARE	AAACCA	946	6	anoxic inducibility
GbbZIP10	TATC-box	TATCCCA	1924	7	gibberellin responsive

GbbZIP10	CGTCA-motif	CGTCA	550	5	MeJA responsive
GbbZIP10	CGTCA-motif	CGTCA	1401	5	MeJA responsive
GbbZIP10	WUN-motif	AAATTTCCT	152	9	wound responsive
GbbZIP10	TGACG-motif	TGACG	550	5	MeJA responsive
GbbZIP10	TGACG-motif	TGACG	1401	5	MeJA responsive
GbbZIP10	MBS	CAACTG	648	6	MYB binding site
GbbZIP10	MBS	CAACTG	1869	6	MYB binding site
GbbZIP10	GARE-motif	TCTGTTG	446	7	gibberellin responsive
GbbZIP10	ABRE	ACGTG	932	5	ABA responsive
GbbZIP10	ABRE	ACGTG	1461	5	ABA responsive
GbbZIP10	G-box	CACGTC	931	6	light responsive
GbbZIP10	G-box	CACGTC	1461	6	light responsive
GbbZIP10	TCA-element	CCATCTTTTT	1179	9	Salicylic acid responsive
GbbZIP10	TCA-element	CCATCTTTTT	1234	9	Salicylic acid responsive
GbbZIP10	TC-rich repeats	ATTCTCTAAC	260	9	defense and stress responsive
GbbZIP10	TC-rich repeats	ATTCTCTAAC	659	9	defense and stress responsive
GbbZIP10	P-box	CCTTTTG	60	7	gibberellin responsive
GbbZIP10	3-AF1 binding site	TAAGAGAGGAA	1813	10	light responsive
GbbZIP18	ABRE	ACGTG	38	5	ABA responsive
GbbZIP18	ABRE	GCAACGTGTC	1571	9	ABA responsive
GbbZIP18	ABRE	CGTACGTGCA	1768	9	ABA responsive
GbbZIP18	ABRE	GCAACGTGTC	1787	9	ABA responsive
GbbZIP18	ABRE	ACGTG	1790	5	ABA responsive
GbbZIP18	ABRE	ACGTG	1825	5	ABA responsive
GbbZIP18	GT1-motif	GGTTAAT	341	7	light responsive
GbbZIP18	GT1-motif	GGTTAA	342	6	light responsive
GbbZIP18	GT1-motif	GGTTAA	705	6	light responsive
GbbZIP18	CAT-box	GCCACT	1081	6	meristem expression
GbbZIP18	G-Box	CACGTT	1789	6	light responsive
GbbZIP18	TC-rich repeats	GTTTTCTTAC	364	9	defense and stress responsive
GbbZIP18	TC-rich repeats	ATTCTCTAAC	556	9	defense and stress responsive
GbbZIP18	G-box	CACGTC	38	6	light responsive
GbbZIP18	G-box	TACGTG	1825	6	light responsive
GbbZIP18	P-box	CCTTTTG	273	7	gibberellin responsive
GbbZIP18	P-box	CCTTTTG	522	7	gibberellin responsive
GbbZIP18	ARE	AAACCA	362	6	anoxic inducibility
GbbZIP18	CGTCA-motif	CGTCA	40	5	MeJA responsive
GbbZIP18	CGTCA-motif	CGTCA	756	5	MeJA responsive
GbbZIP18	CGTCA-motif	CGTCA	1713	5	MeJA responsive
GbbZIP18	RY-element	CATGCATG	1218	8	seed-specific regulation
GbbZIP18	TGACG-motif	TGACG	40	5	MeJA responsive
GbbZIP18	TGACG-motif	TGACG	756	5	MeJA responsive
GbbZIP18	TGACG-motif	TGACG	1713	5	MeJA responsive
GbbZIP18	GC-motif	CCCCCG	1613	6	anoxic inducibility
GbbZIP33	TCA-element	CCATCTTTTT	800	9	Salicylic acid responsive

GbbZIP33	MBS	CAACTG	782	6	MYB binding site
GbbZIP33	GT1-motif	GGTTAA	1522	6	light responsive
GbbZIP33	GT1-motif	GGTTAA	1763	6	light responsive
GbbZIP33	GARE-motif	TCTGTTG	651	7	gibberellin responsive
GbbZIP33	TGACG-motif	TGACG	64	5	MeJA responsive
GbbZIP33	CGTCA-motif	CGTCA	64	5	MeJA responsive
GbbZIP33	MBSI	aaaAaaC(G/C)GTTA	1439	10.5	MYB binding site
GbbZIP33	ARE	AAACCA	445	6	anoxic inducibility
GbbZIP33	ARE	AAACCA	477	6	anoxic inducibility
GbbZIP33	ARE	AAACCA	535	6	anoxic inducibility
GbbZIP33	ARE	AAACCA	1684	6	anoxic inducibility
GbbZIP31	CGTCA-motif	CGTCA	1648	5	MeJA responsive
GbbZIP31	MRE	AACCTAA	1078	7	MYB binding site
GbbZIP31	TGACG-motif	TGACG	1648	5	MeJA responsive
GbbZIP31	TGA-element	AACGAC	1695	6	auxin responsive
GbbZIP31	G-box	ACACGTGT	1171	8	light responsive
GbbZIP31	G-box	CACGTG	1172	6	light responsive
GbbZIP31	G-box	CACGAC	1264	6	light responsive
GbbZIP31	GT1-motif	GTGTGTGAA	1158	9	light responsive
GbbZIP31	CAT-box	GCCACT	1103	6	meristem expression
GbbZIP31	CAT-box	GCCACT	1223	6	meristem expression
GbbZIP31	ABRE	CACGTG	1172	6	ABA responsive
GbbZIP31	ABRE	ACGTG	1173	5	ABA responsive
GbbZIP31	ABRE	ACGTG	1228	5	ABA responsive
GbbZIP31	G-Box	CACGTG	1172	6	light responsive
GbbZIP31	G-Box	CACGTT	1228	6	light responsive
GbbZIP31	MBS	CAACTG	802	6	MYB binding site
GbbZIP31	ACE	CTAACGTATT	616	9	light responsive

Table S4. Correlation analysis of flavoniod content and FPKM of 40 GbbZIPs in 8 tissues

Gene name	Correlation coefficient	P_value
GbbZIP15	0.887436428	0.003271367
GbbZIP08	0.869333882	0.005045068
GbbZIP29	0.79733554	0.017775153
GbbZIP06	0.722402342	0.042963342
GbbZIP12	0.388675781	0.341302565
GbbZIP31	0.329207212	0.425884717
GbbZIP18	0.32013789	0.439493425
GbbZIP11	0.275210356	0.509444332
GbbZIP17	0.251622188	0.547744074
GbbZIP20	0.244132057	0.560115168
GbbZIP36	0.226632349	0.589390569
GbbZIP13	0.221355793	0.598316193
GbbZIP23	0.21892338	0.602445633

GbbZIP16	0.191582134	0.649476433
GbbZIP24	0.11617407	0.784125601
GbbZIP10	0.115058352	0.786162018
GbbZIP39	0.079449199	0.851658437
GbbZIP22	0.031759998	0.940490038
GbbZIP27	-0.032966534	0.938232519
GbbZIP37	-0.067793152	0.873276767
GbbZIP19	-0.075600357	0.858788514
GbbZIP07	-0.094079413	0.8246392
GbbZIP38	-0.187682023	0.656272647
GbbZIP30	-0.250265641	0.549977339
GbbZIP14	-0.260761372	0.532783888
GbbZIP34	-0.274944221	0.509870676
GbbZIP33	-0.330329563	0.424213119
GbbZIP40	-0.330670673	0.423705631
GbbZIP04	-0.357257515	0.38495702
GbbZIP25	-0.397035032	0.330093713
GbbZIP26	-0.407197705	0.316702954
GbbZIP03	-0.424789756	0.294147079
GbbZIP02	-0.443943079	0.270508657
GbbZIP09	-0.456675533	0.25533591
GbbZIP28	-0.460269787	0.251132063
GbbZIP05	-0.483779117	0.224507822
GbbZIP35	-0.498012097	0.209133421
GbbZIP01	-0.527749182	0.178853487
GbbZIP32	-0.546159659	0.161369952
GbbZIP21	-0.726539055	0.041212257

Table S5. miRNA family information of GbmiRNAs targeting GbbZIPs

novel_miRNA	miRNA family	targeting GbbZIPs
novel_miR_1493	MIR1314	GbbZIP01,GbbZIP27
novel_miR_18	MIR397	GbbZIP06
novel_miR_2744	MIR397	GbbZIP06
novel_miR_2184	MIR2950	GbbZIP13
novel_miR_370	MIR2950	GbbZIP13
novel_miR_2537	MIR538	GbbZIP22
novel_miR_2243	MIR159	GbbZIP07
novel_miR_2036	MIR1862	GbbZIP12
novel_miR_1117	MIR2950	GbbZIP13
novel_miR_1291	MIR7717	GbbZIP19
novel_miR_1512	MIR163	GbbZIP21
novel_miR_2434	MIR5512	GbbZIP36
novel_miR_2978	MIR6288	GbbZIP36
novel_miR_3072	MIR159	GbbZIP36

novel_miR_3087	MIR6146	GbbZIP36
novel_miR_3425	MIR5512	GbbZIP36
novel_miR_2847	MIR159	GbbZIP23
novel_miR_775	MIR159	GbbZIP23
novel_miR_2574	MIR2592	GbbZIP31
novel_miR_834	MIR1120	GbbZIP32
novel_miR_3016	MIR5079	GbbZIP33
novel_miR_721	MIR530	GbbZIP33
novel_miR_2905	MIR1122	GbbZIP35
novel_miR_304	MIR1314	GbbZIP37
novel_miR_3054	MIR1314	GbbZIP37
novel_miR_3292	MIR1314	GbbZIP37
novel_miR_443	MIR1314	GbbZIP37
novel_miR_462	MIR1314	GbbZIP37
novel_miR_82	MIR1314	GbbZIP37
novel_miR_109	MIR2950	GbbZIP37
novel_miR_1553	MIR160	GbbZIP40
novel_miR_614	MIR160	GbbZIP40

Table S6. flavoniod content in 8 tissues

Tissues	R	S	IL	ML	M	OS	IF	MF
content	0.13	11.32	75.06	178.71	56.51	12.36	48.68	29.7

Table S8. A local blast of *GbbZIP08* in *A. thaliana* and *M. domestica* bZIP protein database

Rename	ID	(Bits)	Value
AtbZIP56	AT5G11260	132	7.00E-43
MdbZIP67	MDP0000586302	126	5.00E-40
MdbZIP68	MDP0000264514	126	4.00E-39
AtbZIP64	AT3G17609	103	1.00E-31
MdbZIP79	MDP0000219041	99.4	1.00E-28
MdbZIP78	MDP0000279891	90.5	2.00E-24
MdbZIP23	MDP0000834642	80.1	1.00E-21
AtbZIP49	AT3G56660	53.5	1.00E-11
AtbZIP17	AT2G40950	50.4	1.00E-10
MdbZIP30	MDP0000138811	51.6	1.00E-10
MdbZIP29	MDP0000299504	51.6	1.00E-10
MdbZIP12	MDP0000180785	51.2	2.00E-10
MdbZIP11	MDP0000270677	50.8	2.00E-10
AtbZIP61	AT3G58120	46.2	3.00E-09
AtbZIP34	AT2G42380	46.2	3.00E-09
AtbZIP30	AT2G21230	45.8	5.00E-09
AtbZIP28	AT3G10800	44.3	2.00E-08
AtbZIP40	AT1G03970	43.5	2.00E-08

MdbZIP104	MDP0000555457	44.7	2.00E-08
MdbZIP61	MDP0000133698	44.3	4.00E-08
MdbZIP20	MDP0000134936	43.5	6.00E-08
MdbZIP51	MDP0000306302	43.1	7.00E-08
MdbZIP50	MDP0000178326	42.7	1.00E-07
AtbZIP13	AT5G44080	40.8	2.00E-07
AtbZIP37	AT4G34000	40.8	2.00E-07
MdbZIP45	MDP0000701734	41.6	3.00E-07
MdbZIP22	MDP0000247372	40.8	6.00E-07
MdbZIP71	MDP0000479652	40.8	6.00E-07
AtbZIP33	AT2G12900	39.3	7.00E-07
MdbZIP112	MDP0000129203	40.4	7.00E-07
MdbZIP73	MDP0000210251	40.4	7.00E-07
MdbZIP24	MDP0000231542	40.4	7.00E-07
MdbZIP65	MDP0000144105	40.4	7.00E-07
MdbZIP64	MDP0000177486	40.4	7.00E-07
AtbZIP12	AT2G41070	39.3	8.00E-07
AtbZIP66	AT3G56850	39.3	8.00E-07
AtbZIP51	AT1G43700	39.3	9.00E-07
MdbZIP34	MDP0000215106	40	9.00E-07
MdbZIP5	MDP0000273211	40	9.00E-07
AtbZIP32	AT2G12940	38.9	1.00E-06
AtbZIP39	AT2G36270	38.9	1.00E-06
AtbZIP31	AT2G13150	38.5	1.00E-06
AtbZIP69	AT1G06070	38.5	1.00E-06
MdbZIP98	MDP0000602946	40	1.00E-06
MdbZIP37	MDP0000282828	40	1.00E-06
MdbZIP92	MDP0000435971	39.7	1.00E-06
MdbZIP100	MDP0000250967	39.7	1.00E-06
MdbZIP70	MDP0000280559	39.7	1.00E-06
MdbZIP38	MDP0000300820	39.7	1.00E-06
MdbZIP46	MDP0000250947	39.7	1.00E-06
AtbZIP38	AT3G19290	38.5	2.00E-06
AtbZIP27	AT2G17770	37.7	2.00E-06
AtbZIP52	AT1G06850	37.7	2.00E-06
MdbZIP31	MDP0000378041	37.7	2.00E-06
MdbZIP113	MDP0000123107	38.9	2.00E-06
MdbZIP26	MDP0000248567	38.9	2.00E-06
MdbZIP114	MDP0000267964	38.9	2.00E-06
MdbZIP18	MDP0000488746	38.9	2.00E-06
MdbZIP13	MDP0000293847	38.9	2.00E-06
MdbZIP17	MDP0000301399	38.9	2.00E-06
AtbZIP14	AT4G35900	37.4	3.00E-06
AtbZIP59	AT2G31370	37.4	3.00E-06
MdbZIP47	MDP0000706379	38.5	3.00E-06

MdbZIP56	MDP0000740787	38.5	3.00E-06
MdbZIP90	MDP0000296303	38.5	3.00E-06
AtbZIP18	AT2G40620	37.4	4.00E-06
MdbZIP59	MDP0000305387	38.5	4.00E-06
MdbZIP14	MDP0000120158	38.5	4.00E-06
MdbZIP58	MDP0000297791	38.5	4.00E-06
MdbZIP15	MDP0000295681	38.1	4.00E-06
AtbZIP50	AT1G77920	37	5.00E-06
AtbZIP09	AT5G24800	36.6	5.00E-06
MdbZIP97	MDP0000129112	38.1	5.00E-06
AtbZIP35	AT1G49720	36.6	6.00E-06
AtbZIP72	AT5G07160	35.8	7.00E-06
MdbZIP99	MDP0000198495	37	9.00E-06
MdbZIP105	MDP0000120802	37	9.00E-06
AtbZIP29	AT4G38900	36.2	1.00E-05
MdbZIP86	MDP0000307943	37	1.00E-05
MdbZIP84	MDP0000320322	36.6	1.00E-05
AtbZIP70	AT5G60830	35	2.00E-05
MdbZIP109	MDP0000536881	36.2	2.00E-05
AtbZIP05	AT3G49760	33.9	3.00E-05
MdbZIP87	MDP0000905135	35	3.00E-05
MdbZIP28	MDP0000917315	35	3.00E-05
MdbZIP33	MDP0000320524	35.4	3.00E-05
MdbZIP76	MDP0000301884	35.4	3.00E-05
MdbZIP6	MDP0000300532	35.4	4.00E-05
MdbZIP75	MDP0000145555	35.4	4.00E-05
MdbZIP53	MDP0000208334	35	4.00E-05
AtbZIP21	AT1G08320	34.3	5.00E-05
AtbZIP65	AT5G06839	34.3	5.00E-05
AtbZIP54	AT4G01120	33.5	7.00E-05
AtbZIP20	AT5G06950	33.5	7.00E-05
MdbZIP19	MDP0000772665	34.3	8.00E-05
MdbZIP95	MDP0000121258	34.3	8.00E-05
AtbZIP03	AT5G15830	32.3	1.00E-04
AtbZIP26	AT5G06960	32.7	1.00E-04
AtbZIP45	AT3G12250	32.7	1.00E-04
MdbZIP66	MDP0000262210	33.9	1.00E-04
AtbZIP16	AT2G35530	32.3	2.00E-04
AtbZIP63	AT5G28770	32.3	2.00E-04
AtbZIP11	AT4G34590	31.6	2.00E-04
AtbZIP74	AT2G21235	32	2.00E-04
MdbZIP16	MDP0000222114	33.1	2.00E-04
MdbZIP57	MDP0000234166	33.1	2.00E-04
MdbZIP88	MDP0000231274	33.1	2.00E-04
AtbZIP36	AT1G45249	32	3.00E-04

AtbZIP47	AT5G65210	32	3.00E-04
AtbZIP46	AT1G68640	31.6	4.00E-04
MdbZIP93	MDP0000169473	32.3	4.00E-04
MdbZIP96	MDP0000277999	32.7	4.00E-04
MdbZIP52	MDP0000141948	32.3	4.00E-04
AtbZIP42	AT3G30530	30.8	5.00E-04
AtbZIP57	AT5G10030	30.8	6.00E-04
AtbZIP68	AT1G32150	30.4	8.00E-04
AtbZIP08	AT1G68880	29.3	0.001
MdbZIP1	MDP0000197219	31.2	0.001
MdbZIP27	MDP0000185553	31.2	0.001
AtbZIP41	AT4G36730	29.3	0.002
AtbZIP48	AT2G04038	28.5	0.003
MdbZIP81	MDP0000441891	29.6	0.003
MdbZIP80	MDP0000431572	29.6	0.003
MdbZIP103	MDP0000863909	29.3	0.003
AtbZIP15	AT5G42910	28.5	0.004
AtbZIP02	AT2G18160	27.7	0.004
MdbZIP106	MDP0000386314	28.9	0.005
AtbZIP53	AT3G62420	27.3	0.006
AtbZIP07	AT4G37730	27.7	0.007
MdbZIP4	MDP0000251332	28.5	0.007
MdbZIP2	MDP0000249561	28.1	0.007
MdbZIP44	MDP0000200822	28.1	0.008
MdbZIP69	MDP0000636541	28.5	0.008
MdbZIP40	MDP0000205823	28.1	0.008
MdbZIP43	MDP0000140166	28.1	0.008
AtbZIP60	AT1G42990	27.3	0.009
AtbZIP76	AT1G58110	27.3	0.009
MdbZIP36	MDP0000493795	27.7	0.011
MdbZIP35	MDP0000138052	28.1	0.011
AtbZIP22	AT1G22070	26.9	0.012
MdbZIP111	MDP0000545420	27.7	0.012
MdbZIP72	MDP0000448715	27.3	0.013
AtbZIP06	AT2G22850	26.2	0.018
MdbZIP77	MDP0000891899	26.6	0.027
MdbZIP60	MDP0000239026	26.2	0.027
AtbZIP19	AT4G35040	25.4	0.031
AtbZIP55	AT2G46270	25.8	0.031
MdbZIP7	MDP0000174930	26.2	0.041
MdbZIP3	MDP0000265875	25.8	0.042
MdbZIP39	MDP0000521934	25.4	0.048
AtbZIP71	AT2G24340	25	0.049
AtbZIP23	AT2G16770	25	0.05
MdbZIP74	MDP0000270365	25.8	0.054

AtbZIP24	AT3G51960	24.6	0.06
AtbZIP67	AT3G44460	24.6	0.063
AtbZIP77	AT1G35490	24.6	0.064
MdbZIP10	MDP0000190186	25.4	0.073
MdbZIP9	MDP0000893802	25.4	0.073
MdbZIP8	MDP0000898701	25.4	0.073
MdbZIP94	MDP0000261154	25.4	0.087
MdbZIP102	MDP0000286846	25	0.11
MdbZIP91	MDP0000680042	24.6	0.14
AtbZIP79	AT5G04840	23.5	0.15
MdbZIP54	MDP0000159670	24.6	0.15
MdbZIP25	MDP0000275309	24.6	0.17
MdbZIP55	MDP0000176747	24.3	0.2
AtbZIP01	AT5G49450	21.9	0.41
MdbZIP42	MDP0000319187	21.9	0.94
MdbZIP21	MDP0000437680	21.6	1.1
MdbZIP89	MDP0000190277	21.6	1.2
MdbZIP62	MDP0000234798	21.2	1.3
MdbZIP63	MDP0000949327	21.2	1.3
MdbZIP85	MDP0000239688	21.2	2

Table S9. A local blast of *GbbZIP15* in *A. thaliana* and *M. domestica* bZIP protein database

Rename	ID	(Bits)	Value
MdbZIP67	MDP0000586302	133	5.00E-41
AtbZIP56	AT5G11260	131	1.00E-40
MdbZIP68	MDP0000264514	132	8.00E-40
AtbZIP64	AT3G17609	108	6.00E-32
MdbZIP79	MDP0000219041	103	7.00E-29
MdbZIP78	MDP0000279891	94.7	1.00E-24
MdbZIP23	MDP0000834642	82.8	2.00E-21
MdbZIP30	MDP0000138811	58.2	7.00E-12
MdbZIP29	MDP0000299504	58.2	7.00E-12
AtbZIP49	AT3G56660	55.1	3.00E-11
MdbZIP12	MDP0000180785	55.1	7.00E-11
MdbZIP11	MDP0000270677	55.1	7.00E-11
AtbZIP17	AT2G40950	52	3.00E-10
AtbZIP40	AT1G03970	50.8	4.00E-10
AtbZIP09	AT5G24800	47	9.00E-09
AtbZIP13	AT5G44080	45.1	4.00E-08
AtbZIP28	AT3G10800	45.4	4.00E-08
MdbZIP51	MDP0000306302	45.1	6.00E-08
AtbZIP37	AT4G34000	44.7	8.00E-08
AtbZIP72	AT5G07160	42.7	8.00E-08
AtbZIP27	AT2G17770	43.1	1.00E-07

MdbZIP104	MDP0000555457	44.3	1.00E-07
AtbZIP30	AT2G21230	43.5	2.00E-07
AtbZIP33	AT2G12900	42.7	2.00E-07
AtbZIP61	AT3G58120	42.7	2.00E-07
AtbZIP70	AT5G60830	42	2.00E-07
MdbZIP84	MDP0000320322	44.7	2.00E-07
MdbZIP34	MDP0000215106	44.3	2.00E-07
MdbZIP56	MDP0000740787	44.3	2.00E-07
MdbZIP50	MDP0000178326	43.9	2.00E-07
MdbZIP5	MDP0000273211	44.3	2.00E-07
MdbZIP20	MDP0000134936	43.5	2.00E-07
MdbZIP65	MDP0000144105	43.9	2.00E-07
MdbZIP64	MDP0000177486	43.9	2.00E-07
AtbZIP34	AT2G42380	42.4	3.00E-07
MdbZIP24	MDP0000231542	43.9	3.00E-07
MdbZIP61	MDP0000133698	43.5	3.00E-07
AtbZIP38	AT3G19290	42.4	4.00E-07
AtbZIP14	AT4G35900	41.6	5.00E-07
MdbZIP98	MDP0000602946	42.7	5.00E-07
MdbZIP87	MDP0000905135	41.6	5.00E-07
AtbZIP66	AT3G56850	40.8	8.00E-07
AtbZIP12	AT2G41070	40.8	9.00E-07
MdbZIP45	MDP0000701734	42	1.00E-06
MdbZIP100	MDP0000250967	42	1.00E-06
AtbZIP39	AT2G36270	40.4	2.00E-06
AtbZIP52	AT1G06850	40	2.00E-06
MdbZIP97	MDP0000129112	40.8	2.00E-06
MdbZIP18	MDP0000488746	40	3.00E-06
MdbZIP17	MDP0000301399	40	3.00E-06
MdbZIP47	MDP0000706379	40.4	3.00E-06
AtbZIP35	AT1G49720	39.3	4.00E-06
AtbZIP51	AT1G43700	38.9	4.00E-06
AtbZIP50	AT1G77920	38.9	4.00E-06
MdbZIP26	MDP0000248567	40	4.00E-06
MdbZIP22	MDP0000247372	40	4.00E-06
AtbZIP16	AT2G35530	38.9	5.00E-06
AtbZIP20	AT5G06950	38.5	5.00E-06
AtbZIP65	AT5G06839	38.5	6.00E-06
AtbZIP21	AT1G08320	38.5	6.00E-06
MdbZIP13	MDP0000293847	39.7	6.00E-06
MdbZIP6	MDP0000300532	39.3	6.00E-06
MdbZIP31	MDP0000378041	37	6.00E-06
MdbZIP90	MDP0000296303	39.7	6.00E-06
MdbZIP70	MDP0000280559	39.3	7.00E-06
MdbZIP28	MDP0000917315	38.1	7.00E-06

MdbZIP71	MDP0000479652	39.3	7.00E-06
MdbZIP86	MDP0000307943	39.3	7.00E-06
MdbZIP112	MDP0000129203	38.9	7.00E-06
AtbZIP69	AT1G06070	38.1	8.00E-06
MdbZIP73	MDP0000210251	38.9	8.00E-06
MdbZIP33	MDP0000320524	39.3	8.00E-06
MdbZIP53	MDP0000208334	38.9	8.00E-06
MdbZIP99	MDP0000198495	38.9	9.00E-06
AtbZIP74	AT2G21235	37.7	1.00E-05
MdbZIP58	MDP0000297791	38.9	1.00E-05
MdbZIP113	MDP0000123107	38.1	1.00E-05
MdbZIP37	MDP0000282828	38.9	1.00E-05
MdbZIP59	MDP0000305387	38.9	1.00E-05
MdbZIP14	MDP0000120158	38.9	1.00E-05
MdbZIP38	MDP0000300820	38.5	1.00E-05
MdbZIP75	MDP0000145555	38.5	1.00E-05
MdbZIP76	MDP0000301884	38.5	1.00E-05
MdbZIP15	MDP0000295681	38.1	1.00E-05
AtbZIP59	AT2G31370	37.4	2.00E-05
AtbZIP18	AT2G40620	36.6	2.00E-05
AtbZIP32	AT2G12940	36.6	2.00E-05
MdbZIP109	MDP0000536881	37.7	2.00E-05
MdbZIP88	MDP0000231274	37	3.00E-05
MdbZIP95	MDP0000121258	37.4	3.00E-05
MdbZIP105	MDP0000120802	37	3.00E-05
MdbZIP46	MDP0000250947	37.4	3.00E-05
AtbZIP26	AT5G06960	36.2	4.00E-05
AtbZIP45	AT3G12250	36.2	4.00E-05
AtbZIP54	AT4G01120	36.2	4.00E-05
AtbZIP11	AT4G34590	34.7	4.00E-05
MdbZIP92	MDP0000435971	37	4.00E-05
MdbZIP66	MDP0000262210	36.6	5.00E-05
MdbZIP114	MDP0000267964	36.2	5.00E-05
AtbZIP05	AT3G49760	34.3	6.00E-05
MdbZIP93	MDP0000169473	36.2	6.00E-05
AtbZIP29	AT4G38900	35.4	7.00E-05
AtbZIP46	AT1G68640	35	8.00E-05
MdbZIP27	MDP0000185553	36.2	8.00E-05
MdbZIP19	MDP0000772665	35.4	9.00E-05
AtbZIP47	AT5G65210	34.7	1.00E-04
AtbZIP07	AT4G37730	33.9	1.00E-04
MdbZIP96	MDP0000277999	35.4	1.00E-04
AtbZIP15	AT5G42910	33.9	2.00E-04
AtbZIP36	AT1G45249	33.5	2.00E-04
MdbZIP43	MDP0000140166	33.9	2.00E-04

MdbZIP52	MDP0000141948	34.7	2.00E-04
MdbZIP1	MDP0000197219	34.3	3.00E-04
MdbZIP44	MDP0000200822	33.1	3.00E-04
MdbZIP16	MDP0000222114	33.9	3.00E-04
MdbZIP4	MDP0000251332	33.9	3.00E-04
AtbZIP57	AT5G10030	32.7	4.00E-04
MdbZIP69	MDP0000636541	33.5	4.00E-04
AtbZIP63	AT5G28770	32.3	5.00E-04
AtbZIP68	AT1G32150	32	7.00E-04
MdbZIP85	MDP0000239688	33.1	7.00E-04
AtbZIP41	AT4G36730	32	8.00E-04
AtbZIP02	AT2G18160	31.2	8.00E-04
AtbZIP60	AT1G42990	31.2	0.001
MdbZIP35	MDP0000138052	32.3	0.001
MdbZIP36	MDP0000493795	31.6	0.002
MdbZIP54	MDP0000159670	31.6	0.002
MdbZIP111	MDP0000545420	31.2	0.002
MdbZIP57	MDP0000234166	31.2	0.002
AtbZIP06	AT2G22850	29.6	0.003
MdbZIP40	MDP0000205823	30.4	0.003
AtbZIP53	AT3G62420	29.3	0.004
AtbZIP48	AT2G04038	29.3	0.004
MdbZIP81	MDP0000441891	30.4	0.005
MdbZIP80	MDP0000431572	30.4	0.005
MdbZIP60	MDP0000239026	29.6	0.005
AtbZIP22	AT1G22070	29.3	0.006
AtbZIP23	AT2G16770	28.9	0.006
MdbZIP2	MDP0000249561	29.3	0.006
MdbZIP72	MDP0000448715	28.9	0.007
MdbZIP39	MDP0000521934	28.9	0.008
MdbZIP7	MDP0000174930	28.9	0.013
AtbZIP79	AT5G04840	28.1	0.014
MdbZIP102	MDP0000286846	28.9	0.017
AtbZIP42	AT3G30530	26.9	0.02
AtbZIP19	AT4G35040	27.3	0.022
MdbZIP74	MDP0000270365	28.1	0.024
MdbZIP77	MDP0000891899	27.7	0.026
MdbZIP3	MDP0000265875	27.3	0.029
AtbZIP24	AT3G51960	26.6	0.03
MdbZIP55	MDP0000176747	27.7	0.034
MdbZIP25	MDP0000275309	27.7	0.035
MdbZIP106	MDP0000386314	26.9	0.061
MdbZIP42	MDP0000319187	26.9	0.063
AtbZIP77	AT1G35490	25.8	0.072
MdbZIP10	MDP0000190186	26.6	0.072

MdbZIP9	MDP0000893802	26.6	0.072
MdbZIP8	MDP0000898701	26.6	0.072
MdbZIP62	MDP0000234798	25.4	0.12
MdbZIP21	MDP0000437680	25.4	0.12
MdbZIP63	MDP0000949327	25.4	0.12
MdbZIP91	MDP0000680042	25.8	0.12
AtbZIP67	AT3G44460	25	0.14
MdbZIP94	MDP0000261154	25.8	0.14
AtbZIP03	AT5G15830	22.7	0.61
AtbZIP71	AT2G24340	22.3	0.84
AtbZIP08	AT1G68880	19.6	5
MdbZIP103	MDP0000863909	20.4	5.8
